# Supplementary material for: Tracking single hiPSC-derived cardiomyocyte contractile function using CONTRAX an efficient pipeline for traction force measurement
Source: Nat Commun. 2024 Jun 26;15:5427. doi: 10.1038/s41467-024-49755-3 (PMC11208611; doi:10.1038/s41467-024-49755-3)
Supplement: Supplementary file 1 — Supplementary Information [file 41467_2024_49755_MOESM1_ESM.pdf]

SUPPLEMENTARY INFORMATION

Tracking single hiPSC-derived cardiomyocyte contractile function using CONTRAX an efficient pipeline for traction force measurement

Supplementary Methods

CONTRAX Software: detailed description and workflow

Cell Locator

Description

The CONTRAX *Cell-Locator* module is a MATLAB-based graphic user interface (GUI) that enables the automated selection and localization of micropatterned hiPSC-CMs. We developed it to increase the throughput of video acquisition by removing the bottleneck created by the manual localization and identification of single contracting hiPSC-CMs (**Figure 1.a**). Using a sequence of image-processing steps including thresholding, edge detection, masking, and filtering, thousands of relevant single hiPSC-CMs are automatically identified and located within tens of seconds. User-defined morphological search criteria filter subpopulations and exclude alien objects; contraction detection and fluorescence expression level can be used as additional filtering criteria. Automation using such criteria increases the yield of TFM acquisition by only selecting contracting cells or identifying a cell subtype based on the level of expression of a specific protein using a live-cell fluorescent reporter or dye. The absolute positions of the cells on the microscope stage are calculated from their respective locations within a tile image and the morphology of each cell is analyzed in terms of area, width, length, aspect ratio, and main elongation axis orientation.

As such, this software leads to rapid and unbiased selection and morphological analysis of single cells, and—critically—enables the subsequent automated acquisition of TFM video recordings. The software's output is of broad applicability to the analysis of cell morphology in any given cell population and single-cell detection, and not solely limited to the analysis of contracting hiPSC-CMs via TFM.

Input – Output

The *Cell-Locator* module takes as input images of file types *.tiff* or *.czi*, forming the tiles of the survey. If contraction detection or fluorescence intensity is desired for detection or analysis, then time stacks or multichannel stacks can be provided as input. For *.tiff* images, the associated stage position must be provided, which can be obtained from the microscopy software used for survey acquisition. Any desired magnification and illumination technique (*e.g.,* bright field, phase, or fluorescence microscopy) can be used, and the corresponding pixel-to-micron conversion must be provided. The algorithm calculates the centroid position of the cells. The output is translated from pixels to microns using the micron/pixel conversion for the objective used. The position of a cell in an image is then translated into the absolute stage position using the stage position of the image. Once the stage position is known, it is possible to use any magnification to image a given cell. This requires well calibrated stage and objective turret alignment, or the application of an offset to all position to compensate. When starting a high-magnification acquisition, the user measure this offset and apply the necessary offset compensation to the rest of the positions in the list. The Automated TFM Acquisition allows to perform an autofocus at each cell position, which is recommended to correct for the focal plane offset often occurring with a change of objective and magnification on most microscopes.

The *Cell-Locator* module outputs: 1) a position list for subsequent automated acquisition of TFM videos at higher magnification; and 2) a results file with single cell-level morphological information and population statistics. The x-y-z positions of the cells are returned as a position list *.pos* file for *Micro-Manager* (The Open Source Microscopy Software: https://micro-manager.org) or as a stage mark list *.czstm* file for *Zen Blue 2.6* (Carl Zeiss Microscopy: https://www.zeiss.com/microscopy/us/products/microscope-software/zen.html); results are exported to a *.csv* spreadsheet.

Workflow

After providing the input data, the user is requested to define search criteria for the ranges of area, aspect ratio, and orientation in which cells are to be identified and selected. Before automatic processing of the entire stack of images of the survey, the search criteria can be quickly tested and refined using a single image. The software then automatically detects thousands of single cells matching the search criteria. If desired, the detected cells can be reviewed and excluded manually, and additional cells can be manually added. Identified cells are marked in each tile image and a zoomed-in image of each cell with its detected outline is displayed on the side of the screen with the corresponding morphological parameter for easy and rapid review.

If contraction detection is desired, then time-stack images can be provided as input and a built-in algorithm detects cell contraction for further filtering. To achieve this detection, the image survey can be performed using a short time lapse of >2 frames at a framerate sufficient to slightly oversample a contraction cycle. For example, 4 frames at 166-ms intervals maximizes the chance of acquiring at least a part of the contracted or relaxed state of a cell beating at 1 Hz. Our time-efficient algorithm calculates the z-project standard deviation for each pixel, as a measure of the local variation over time. It then calculates the average value of this standard deviation within a cell area and determine whether the local variation over time within the cell area is above a user-chosen threshold compared to the rest of the frame (for example how the average time standard deviation of a cell compares to the average time standard deviation in the image). A slider adjusts the threshold value and the software dynamically displays the threshold mask over the image, enabling easy identification of an optimal threshold for a given image.

If fluorescence-based selection is desired, then multichannel stack images can be provided as input and a built-in algorithm filters cells based on fluorescence intensity. In this case, the image survey should be performed with the appropriate fluorescent channel in addition to the transmitted light imaging. The average intensity of fluorescence is calculated within each cell area and a slider enables the user to adjust the detection threshold (similar to contraction detection), thereby enabling selection of cells based on fluorescence intensity.

Automated TFM Acquisition module

Description

The *Automated TFM Acquisition* script automates the acquisition of TFM videos of single contracting hiPSC-CMs. We developed it to address the throughput bottleneck created by the manual acquisition of live videos of previously identified single contracting hiPSC-CMs. *Automated TFM Acquisition* contributes to increasing the throughput of the workflow and reducing the burden on user/s by automating the acquisition of video recording using the cell position list from the *Cell-Locator* module (Figure 1.a). Two versions were developed, one each for *Micro-Manager* and for *Zen Blue 2.6*, because neither software enables a direct acquisition sequence in hierarchical order: *Position (with autofocus at each position)* □ *Channel* □ *Time (video streaming)*. The scripts were written in the *Beanshell* scripting language in *Micro-manager*, and in *Python* in *Zen*. Both versions are fully editable and can be rapidly adapted to fit specific user requirements.

Input–Output

Automated TFM Acquisition takes the cell positions list from the *Cell-Locator* module as input; these positions are loaded as *Stage Position List* in *Micro-Manager* and as *Stage Mark* in *Zen*. The script automates acquisition by using the acquisition parameters (such as exposure time) that the user defines in the microscopy software during experimental setup. During execution, the script loops through the positions list to acquire videos of each hiPSC-CM, first using transmitted light and then using the fluorescence channel corresponding to the fluorescent microspheres (Figure 1.b).

The software outputs videos saved in 8-bit **.tiff* format directly in order to minimize data volume or as *.czi* files in *Zen*. Each file name retains the identity of each cell for easy pairing of single-cell data in time-course experiments.

Workflow

After loading the position list from the *Cell-Locator* module and setting up the experimental parameters in the microscopy software, the user executes the *Automated TFM Acquisition* script. If the culture plate has already been returned to the incubator, it can be loaded again onto the microscope stage. In this case, the cell position list may be off by a fixed offset in x, y, or z; the user should check for an offset, and correct it, within the microscopy software. During automated acquisition of a large number of cells, and due to possible unevenness of the hydrogel surface,

we recommend that the user includes a software autofocus step at each cell position (this is an option in *Automated TFM Acquisition*) to ensure adequate focus. In addition, a focus offset can be programmed to adjust for differences in focal plane between bright field and fluorescence microscopy. Accurate TFM measurements require a good focus position at the top surface of the hydrogel (in direct contact with the cells) when measuring the displacement of fluorescent microspheres.

An additional version of this script yields semi-automated acquisition, making it possible for the user to skip positions or to navigate along the position list manually, with rapid keyboard strokes. This semi-automation helps reduce the overall experimental time at later acquisition timepoints during a time course, as many cells may have detached or died by these later timepoints.

Streamlined TFM

Description

Streamlined TFM is a MATLAB-based GUI for analysis of TFM video data. It addresses the throughput bottleneck created by the computationally- and user-intensive process involved in the processing of TFM data. The *Streamlined TFM* contributes to increasing the throughput of the workflow by reducing the need for user input during data computation and analysis and by speeding up the computation by exploiting parallel processing and efficient data handling (**Figure 1.c**). The *Streamlined TFM* was developed building upon the work of Ribeiro et al.[1] as the base code architecture and TFM algorithm implementation. Details of the calculations and formulas for the measured parameters can be found in Ribeiro et al.[1]

Streamlined TFM offers numerous features in addition to significant streamlining and more efficient computing. Of notable importance are two algorithms for automated cell outlining and automatic contraction peak analysis. Both these algorithms reduce the amount of user input (mouse clicks) by at least an order of magnitude, save a considerable amount of time in terms of data pre/postprocessing, and, importantly, reduce the risk of user bias. Indeed, defining cell outlines is an important step for unconstrained TFM analysis (Figure 1.c) and manual contouring of hundreds of single cells is a very tedious and labor-intensive task that may introduce user-to-user variability.

To solve these problems, our algorithm identifies cell contours through a series of image-processing steps that detect both weak and strong edges despite the presence of image artifacts or fluorescent microspheres visible as black dots in bright field images. While it would also be possible to use fluorescent markers to segments cells, our aim was to keep as many fluorescence channels available as possible for other analyses and to minimize the necessary preparation steps and potential (photo-)toxicity. We therefore focused on outline detection in transmitted light images. The second algorithm speeds the statistical analysis of cell contractile output through the automated analysis of a sequence of contraction peaks. Following TFM computation, the 2-dimensional traction stress data are averaged or integrated depending on requirements for the parameters of interest within the cell area to yield a 1-dimensional time trace of contractile parameter such as total contractile force, contraction velocity, or strain energy (Figure 1.c). In each trace, several contraction peaks must be analyzed to extract the relevant contractile parameters, such as contraction force amplitude, maximum contraction velocity, or maximum relaxation velocity. Our algorithm automates this task, alleviating the need for manual selection of individual peaks. Using a series of data-processing steps, including filtering in the Fourier space and auto/cross-correlations, individual peaks are automatically identified, precisely localized, extracted, aligned, and averaged in each signal trace (Figure 1.c). From this force-versus-time trace, the algorithm identifies the peak and baseline and computes metrics including peak amplitude, duration, mid-peak duration, maximum and minimum derivatives (for example contraction and relaxation velocities), frequency, and integral under the curve (impulse). The computation of the baseline for the most relaxed cell state is of high importance as the traction stress measurement is prone to noise that often results in non-zero values for the parameters calculated from scalar values of the displacement or stress field. Each metric is accompanied by its standard deviation. For example, deviation from the beating frequency is a measure of the rhythmicity of beating. We validated our code with a TFM Benchmarking Model, which we made available in Supplementary information and as preprint as a tool independent of `CONTRAX`.[2] Other model have been recently developed more recently.[3,4]

Input–Output

The inputs for *Streamlined TFM* are video recordings of fluorescent microspheres displaced by contracting hiPSC-CMs and at least one brightfield image of the cell for outlining. File

formats can be *.tif*, *.avi*, or *.czi*. For *.tif* and *.avi* files, the corresponding frame rate and pixel-to-micron conversion must be provided; for *.czi* files, this information is automatically extracted from the metadata.

Streamlined TFM outputs a spreadsheet file containing the summary of the statistical information for the batch of cells analyzed, as well as a data folder for each cell, containing data details, cell outline mask, and images of the contraction traces.

Workflow

The first step after importing the input data is to verify the video parameters and to define the cell outlines. This definition can be done manually or automatically using our new cell outlining algorithm. To automatically detect cell outline, edge detection was performed using Matlab built in `edge` function using the "Canny" methods and sensitivity thresholds of [0.001,.4] to ignore edge that are not stronger than these threshold values. It is possible that image quality may vary depending on the imaging setup used, in which case the threshold values may need adjustment. However, in our hands, these values proved robust with images of varying qualities. Then the following steps are followed:

- The function `adapthisteq` was used to enhances the contrast of the grayscale image by transforming the values using contrast-limited adaptive histogram equalization (CLAHE)
- The function `imbilatfilt` was applied as an edge-preserving Gaussian bilateral filter to the grayscale or RGB image, with a calculate degree of smoothing based on the image two-dimensional standard deviation, and with a standard deviation of the smoothing kernel that is also calculated from the image spatial resolution.
- The function `edge` was applied to detect the cell edges using the "Canny" methods and sensitivity thresholds of [0.001 0.5] to ignore edge that are not stronger than these threshold values.
- The resulting binary image was then dilated to connect the detected edges and filled. The results binary image is then eroded by a similar factor.
- The resulting binary objects are then filtered to exclude small object resulting from noise or strongly showing beads and the convex hull was taken to obtain a single object from remaining individual objects.

- The resulting image was then used to refined the edge detection using the function `activecontour` with 'Chan-Vese' method, a maximum 100 iteration and a 'SmoothFactor' of 3.
- The contour was then smoothened once more with an erode and dilate steps

Alternatively, if a cell outline mask was previously defined, then the user is prompted to automatically import this mask when loading the data. Cell outline masks can also be imported manually later. The user can then define a cropping mask and modify the parameters of the analysis mask (yellow dashed line in Figure 1.b and Figure 1.c), which is defined by anisotropic expansion of an ellipse fitting the cell outline. Finally, the user decides whether cropping, binning, and denoising should be performed. Each of these operations can significantly shorten the processing time and reduce noise in the output data, but should be carefully considered and reported if used, as they may introduce a consistent bias into the results depending on image quality. The *TFM Benchmarking Model* (see Supplementary Methods in Supplementary information) is a useful tool for exploring the impact of these settings.

The second step measures the deformation induced in the hydrogel by contracting hiPSC-CMs by tracking the displacement of the fluorescent microspheres using digital image correlation (DIC). In this step, the user should verify that adequate DIC parameters are used; again, the *TFM Benchmarking Model* is helpful. After DIC computation, the user must confirm or select a correct reference frame for the contracted and relaxed state of the cell, save the data, and proceed to the next step.

The third step computes the traction stresses from the strain fields through Fourier Transform Traction Cytometry (FTTC). This step does not require specific user input, but for the choice of analysis to perform (constrained or unconstrained analysis). The regularization parameter, which is crucial for TFM computation, is calculated automatically using L-curve corner detection.[1,5]

Finally, the results panel displays the computed time traces for each parameter. While the automated peak analysis automatically analyzes each trace parameter and saves them in output files, the user can manually confirm or correct individual parameters, which are then saved in a separate tab in the spreadsheet. The user can select flags and add comments to specific cells for downstream analysis. Details of the calculations can be found in Supplementary information.

Contractile parameters measurement and calculations

The description that follows is adapted from Ribeiro *et al* and significantly modified and augmented.[16]

Using bright-field videos of single beating hiPSC-CMs, a region of interest (ROI) around the borders of cells to analyze and the average displacement within this ROI (Figure 1.b) was measured. A reference frame, representing a relaxed state of the cell, was used to compute the average displacement within the ROI for each frame relative to this reference frame using digital image correlation and particle image velocimetry.11, 12 Cross-correlation was carried out using Ncorr within MATLAB 15, using sub-blocks of the image with a size and spacing determined by user defined parameters matched to the image characteristics, i.e. magnification, beads density, contrast, noise.

The average displacement in the ROI is defined as:

$$d(t_k) = \frac{1}{N}\sum_{k=1}^{N}\sqrt{u_{k,x}^2 + u_{k,y}^2}, \ (1)$$

where $k = 1,..., N$ corresponds to the frame number in the video and (u_x , u_y) are the displacement vector at each pixel. The maximal contraction displacement was defined as the total distance between the fully relaxed and fully contracted states of the cell's contractile cycle. This distance was calculated by subtracting the minima from the maxima of $d(t_k)$. Minima of $d(t_k)$ represents the detected noise, which remains approximately constant in the absence of drift or artefacts.

Maximal contraction displacement is defined as:

$$d_c = \frac{1}{m}\sum_{i=1}^{m}\max_i\big(d(t_k)\big) - \frac{1}{n}\sum_{j=1}^{n}\min_j\big(d(t_k)\big)\ . \ (2)$$

Maxima and minima were defined by m and n. $\max_i$ and $\min_j$ are the local maximum and minimum values, respectively, for each contraction cycle.

The average velocity of movement within the ROI was calculated using the first derivative of the mean movement as:

$$V(t_k) \approx \frac{\Delta d}{\Delta t} = \frac{d_{k+1} - d_{k-1}}{t_{k+1} - t_{k-1}}, \ (3)$$

where $d_k = d(t_k)$. The maximal velocity of contraction (V_c) and the maximal velocity of relaxation (V_R) were extracted as:16

$$V_C = \frac{1}{m}\sum_{i=1}^{m}\max_i(V(t_k)); \ V_R = \frac{1}{n}\sum_{j=1}^{n}\min_j(V(t_k)). \ (4)$$

where, m and n are the number of maxima and minima, respectively, and max_i and min_j are the local maxima and local minima, respectively, for each contraction cycle.

The beating rate (br) is defined as the number contractile cycles per unit of time. Two approaches are used to determine br from the curve $d(t_k)$: discrete Fourier transformation of $d(t_k)$ reveal the dominant frequency peaks; or calculation of br as the inverse of period (T) manually defined as the time between adjacent peaks of on the $d(t_k)$ curve in the time domain, calculated as: br $= (\frac{1}{m}\sum_{i=1}^{m} T_i)^{-1}$. Both approaches deliver the same result, although the second approach offers higher flexibility when $d(t_k)$ has high arrhythmicity.

To determine the duration of each contraction, the time between highest contraction velocity and highest relaxation velocity was calculated using each adjacent maximum and minimum of $\mathrm{V}(t_k)$ and averaged for all contraction cycles:16

$\hat{t} = \frac{1}{m}\sum_{i=1}^{m} \| t_k | \max_i V(t_k) - t_k | \min_i V(t_k) \|$. (6)

Ideally, the time of each contractile cycle could be calculated from $d(t_k)$ However, this approach is difficult because the exact beginning and end of each contraction curve are often hard to select.

Displacement fields for each frame were calculated with respect to a reference frame ($\mathrm{frame_{ref}}$), and the choice of $\mathrm{frame_{ref}}$ is critical for the shape of d-curves and V-curves. The selected $\mathrm{frame_{ref}}$ is not necessarily the first frame of the video, but instead a frame that showed the cell in its most relaxed state. The selection of a reference $\mathrm{frame_{ref}}$ was automated, by first selecting the first frame of the video as initial guess and compared with other frames to select the one that best satisfied the following criteria:

maximize $[m(\mathrm{frame_{ref}}) = \Delta d \approx d_i(t_{\mathrm{frame_{ref}}})_{\max} - d_k(t_{\mathrm{frame_{ref}}})_{\min}]$, (12)

minimize $[n(\mathrm{frame_{ref}}) = \sum_{i=1}^{N}(d_i(t_{\mathrm{frame_{ref}}}) \ )]$ (13)

The first criterion ensures a maximal difference between the maximum and minimum points of the displacement curve. However, the solution for this criterion can result in either the desired $\mathrm{frame_{ref}}$ where the cell is in its most relaxed state, or a $\mathrm{frame_{ref}}$ where the cell is in its most contracted state instead. The second criterion selects a $\mathrm{frame_{ref}}$ that minimizes the area under the displacement curve. These two criteria were enough to automatically identify $\mathrm{frame_{ref}}$ as a frame where the cell is in its most relaxed state. Once frameref was selected, all displacements in regions within the ROI were calculated relative to $\mathrm{frame_{ref}}$.

Traction force microscopy and phenotypes of mechanical output

CONTRAX estimates the forces generated by hiPSC-CMs by traction force microscopy (TFM). TFM estimates forces generated by adherent cells on deformable substrates through two steps:[16,17] i) measuring substrate deformation induced by cell-generated traction, and ii) deriving forces from substrate deformations while considering the Young's modulus (E) and Poisson's ratio (υ) of the substrate.

Dispersed fluorescent microbeads within polyacrylamide hydrogels allows quantification of their displacement during contractions of hiPSC-CMs to track cell-induced deformations of polyacrylamide substrates. Video of cells beating, deforming the hydrogel and thereby moving the embedded microbeads were acquired and submitted to cross-correlation particle tracking as detailed in the beginning of the previous section. Measurement of the displacement of microbeads yields the displacement curve d(t), the maximal velocity of contraction (V_C), the maximal velocity of relaxation (V_R), the beat rate (br), and the time between each adjacent maximum and minimum on the velocity plot ($\hat{t}$).

After determining the displacements of moving microbeads from the videos, the traction stresses $\boldsymbol{\sigma}$ associated with each displacement vector of the surface is computed and the force **f** field and its norm f for each stress vector is calculated using traction force microscopy. The total force F is integrated over the ellipse encompassing each cell area to compute the total amount of force ($\sum f$) that each cell can generate on its extracellular environment during each contractile cycle.[6] Calculating σ and f for each frame yield to plots of $\sum \sigma$ and $\sum f$ as a function of time and relative to a $frame_{ref}$ that is selected to correspond to the relaxed state of a beating cell using the same criteria as for bright-field videos of beating cells, but applied to videos of moving microbeads instead, as explained above. The total contractile power (P) is calculated by multiplying F by the velocity at each point and time point and summed to obtain to the total contractile power ($\sum P$). From the $P(t)$ curve, the maximal power of contraction (P_C) and the maximal power of relaxation (P_R) are extracted.

Two traction force microscopy approaches exist to analyze traction stresses: constrained and unconstrained traction force microscopy. Both approaches were initially developed to quantify the forces of cell adhesion to deformable substrates.[16] Butler and colleagues have shown that defining the deformed region of the gel is key for quantifying cell adhesion forces to exclude erroneous solutions from edge effect and from noise.[16] The constrained approach only accounts

for the generated forces within the area occupied by the cell. In contrast, the unconstrained approach accounts for tractions within and outside of the area occupied by the cell.

In CONTRAX both constrained and unconstrained Fourier-based traction force microscopy can be performed. A user tutorial on how to use the graphical user interfaces is available on our GitHub. Both methods yield surface stresses field ($\boldsymbol{\sigma}$) that are converted to absolute values of traction forces (*F*); we sum the values of ($F(\sum f)$) by integrating all values of f over the respective areas where cells generate contractile forces. In our analysis, we used unconstrained traction force microscopy. CONTRAX assumes by default that polyacrylamide substrates are used with a Poisson ratio of 0.4521 and a material stiffness of 10 kPa,[6] although these values can be changed in the code or directly in the GUI interface.

Traction force microscope uses continuum-mechanics equations for linear elastic materials, which are described through force equilibrium conditions,

$\sigma_{ji,j} + f_i = 0$, (14)

the material constitutive relations,

$\sigma_{ji} = \frac{E}{1+\upsilon}[\varepsilon_{ij} + \frac{\upsilon}{1-2\upsilon}\varepsilon_{kk}\boldsymbol{\delta}_{ij}]$, (15)

and kinematic equations,

$\varepsilon_{ij} = \frac{1}{2}(u_{i,j} + u_{j,i})$, (16)

where $\boldsymbol{\sigma}$ is the stress tensor, **f** is a force of external origin, $\boldsymbol{\varepsilon}$ is the linear strain tensor and **u** is the displacement field. E is the Young's modulus of the polyacrylamide substrate and υ is its Poisson's ratio. E and υ are constants that depend on the properties of the deformable material. For polyacrylamide substrates, E is tunable in the kPa range[20] and υ is ~0.45 for thin polyacrylamide sheets used for cell culture.[21] Equations 14-16 correspond to 15 equations with 15 unknowns that are expressed in a condensed form using the Einstein summation convention and the Kronecker delta δ_{ij} ($\delta_{ij} = 0$ if i ≠ j, $\delta_{ij} = 1$ if i = j) . Note that $\boldsymbol{\delta}_{ij}$ is not related to the variable defined in Equations 9 and 10, but uses the same notation. These equations are valid assuming that the strains are small and linear and that the polyacrylamide substrate has homogeneous properties and behaves as an elastic solid.[22] These assumptions satisfy the need for geometric linearity of strain and material linearity of the substrate. By combining the governing equations, the balance of internal forces described in equation 14 can be written as a partial differential equation for the displacement vector field:

$$\frac{E}{2(1+v} [(u_{j,ij} + u_{i,jj}) + \frac{2v}{1-2v} u_{k,ij}\delta_{ji}] \quad + \mathbf{f}_i = 0. \ (17)$$

Cell-generated deformations of a polyacrylamide hydrogel are assumed to occur in a semi-infinite elastic medium with a planar traction distribution on its surface. Specifically, for a semi-infinite elastic medium bounded by a planar surface at z=0, we used a derivation of the Boussinesq solution to describe the deformations of the medium under the influence of a concentrated point force **f** applied to the surface. This relationship between local displacement **u** **f** can be represented using the Green's tensor ***G*** as

$$\boldsymbol{u}_i = \boldsymbol{G}_{ij}(x, y, z)\mathbf{f}_j. \ (18)$$

All local displacements were further assumed to occur in-plane, $\mathbf{u} = (u_x \ u_y)^T$. Tractions stresses were also assumed to have a zero normal component to the displacement plane, $\mathbf{f} = (F_x \ F_y)^T$. v was assumed to be close to 0.5 for polyacrylamide hydrogels.[16] The problem can therefore be reduced to x and y coordinates to represent the two-dimensional movement of fluorescent microbeads being deformed due to cellular traction. Under these assumptions:

$$\boldsymbol{G}_{ij} = \frac{1+v}{\pi E} \frac{1}{r^3} \begin{bmatrix} (1-v)r^2 + vx^2 & -vxy \\ -vxy & (1-v)r^2 + vy^2 \end{bmatrix}, \ (19)$$

where $r = \sqrt{x^2 + y^2}$and the off-diagonal elements are corrected with a minus sign.[17] To calculate cell- generated traction forces **T**(x,y), Equation 17 can be represented as:

$$\mathbf{u}_i = \iint \mathbf{G}_{ij}(x - x', y - y')\mathbf{T}_i(x', y')dx'dy'. \ (20)$$

Equation 19 corresponds to a spatial convolution of **G** and **T**, which Butler and colleagues first denoted as $\mathbf{u} = \mathbf{G} \otimes \mathbf{T}$,25 and represents displacement as a function of known traction forces. To determine **T** as a function of **u**, Equation 20 must be inverted, which requires transformation into the Fourier space, as **G** is not diagonal. Using the convolution theorem,[16], the problem becomes $\tilde{\mathbf{u}}(\mathbf{k}) = \tilde{\mathbf{G}}(\mathbf{k})\tilde{\mathbf{T}}(\mathbf{k})$ and the transformed matrix $\tilde{\boldsymbol{G}}$ is expressed as:

$$\tilde{\mathbf{G}}_{ij} = \frac{1+v}{\pi E} \frac{2\pi}{k^3} \begin{bmatrix} (1-v)k^2 + vk_y^2 & -vk_x \ k_y \\ -vk_x \ k_y & (1-v)k^2 + vk_x^2 \end{bmatrix}, \ (21)$$

where $\mathbf{k} = \sqrt{k_x^2 + k_y^2}$ and $\mathbf{k}_i$ represent wave vectors.

The traction forces were then computed using the inverse Fourier transformation,

$$\mathbf{T} = \mathcal{F}^{-1}\{\tilde{\mathbf{G}}^{-1}\tilde{\mathbf{u}}\}. \ (22)$$

As detailed by Butler and colleagues,[16], to solve this equation, the Nyquist frequency limitation is solved by setting the off-diagonal elements of Equation 20 to 0, if at a Nyquist frequency in x-axis or y-axis. The displacement values resultant from noise were also filtered while calculating traction force. In summary, to filter noise without altering signal, smoothing was achieved with zero-order Tikhonov regularization, which was initially adapted by Sabass and colleagues[17] while solving this Fourier transformation problem. Sabass and colleagues altered Equation 21 into

$$\mathbf{T} = \mathcal{F}^{-1}\{(\widetilde{\mathbf{G}}^T\widetilde{\mathbf{G}} + \lambda^2\widetilde{\mathbf{H}})^{-1}\widetilde{\mathbf{G}}^T\widetilde{\mathbf{u}}\}. \quad (23)$$

The regularization parameter λ determines the amount of the solution that originates from the regularization parameter relative to the data. $\mathbf{H}$ corresponds to the identity $\|_2$ for a zero-order regularization.

Once the stress of σ is calculated, the conversion of σ to f was carried out for each quadratic element of the traction grid that results from submitting videos of moving microbeads to the image analysis routine that defines traction force microscopy. The σ was multiplied by the area of each respective grid element. For constrained force calculation, f is integrated within the ROI defined by the cell borders. For unconstrained measurements, an extended ellipse with the same center of mass as the ROI is calculated and used to integrate f within the region delimited by this extended ellipse to determine F = $\sum f$. This approach allows the analysis of one cell at a time within a video of multiple cells in an array; it also does not quantify noise in regions of the substrate that are far from the cell where negligible deformation and stress occurs. The area of the extended ellipse relates to the area of the ROI as

$$A_{\text{ellipse}} = n \cdot A_{\text{ROI}}. \quad (24)$$

To calculate the extended ellipse, we set the constant n to values between 2 and 3 (default) and set the orientation of the major axis (a) and that of the minor axis (b) of the ellipse to match the orientation of the major and minor axes of the ROI (Figure 1.e), respectively. Therefore,

$$a_{\text{ell}} = m \cdot \sqrt{n} \cdot a_{\text{ROI}},$$

and

$$b_{\text{ell}} = \frac{1}{m} \cdot \sqrt{n} \cdot b_{\text{ROI}}.$$

where m is a factor controlling the eccentricity of the scale ellipse to avoid unwanted over-elongation of the scale ellipse. In `CONTRAX`, m is set by default to 0.75.

The force is then computed as

$$\mathbf{F}(\vec{\mathbf{r}}) = \sum_{\vec{\mathbf{r}}} \boldsymbol{\sigma}(\vec{\mathbf{r}})$$

Unconstrained Traction Force Microscopy

In the unconstrained analysis, a Fourier transform is applied to each displacement map. Then, for each wave number, tractions are set at *f*=0 to 0 (Equation 13), **G** is computed according to Equation 20, and the diagonal elements are set to 0 at Nyquist frequency.25 Regularization is computed as defined in Equation 23. The regularization parameter λ is computed for the first frame of displacing microbeads in a video using the Regutools toolbox in MATLAB.27 Because noise does not vary within a video, this calculated λ is applied to the analysis of subsequent frames. Independent value of λ is calculated for each analyzed video. An alternative could be to calculate a single λ for a batch of video, to standardize the analysis across an experiment. After calculating stress values in the Fourier space for each pixel, the stresses are transformed back to the real space to obtained a map of stresses for each frame.

Accurately calculating λ is key for generating reliable solutions because Equation 23 represents an ill-posed problem in which arbitrarily small perturbations of input data can lead to arbitrarily large perturbations of the solution. Calculation of via the Regutools toolbox27 solves an ill-posed problem defined as $\mathbf{Ax} = \mathbf{b}$ that satisfies the following criteria:

1. the singular values **A** of tend to zero, and
2. the ratio between the smallest non-zero values of **A** is large.

A side constraint ($\Omega(\mathbf{x})$) was introduced to minimize the norm $||\mathbf{Ax} = \mathbf{b}\ ||$while minimizing $\Omega(\mathbf{x})$. In the Tikhonov regularization approach, λ represents the weighing between the data and $\Omega(\mathbf{x})$:

$min_x||\mathbf{Ax} = \mathbf{b}\ ||^2 + \lambda^2\Omega(\mathbf{x})^2$. (27)

Large values of λ cause an excessive level of smoothing, while small λ values increase the weight of noise pronounced in $\mathbf{Ax} = \mathbf{b}$. The ability of this approach to calculate a suitable λ with the L-curve criterion as been validated.28

Constrained Traction Force Microscopy

In constrained traction force microscopy analysis,19 the same inputs as for the unconstrained analysis are required, as well as additional information about the ROI of the cell. Stresses are then calculated as detailed for the unconstrained calculation and a new traction field is defined

by setting the tractions outside of the ROI to zero. The displacement field is calculated that corresponds to this new traction field and experimental values of displacement inside the ROI are replaced by the calculated displacement values.19 The calculation of stress from the displacements is then reiterated within the ROI to calculate new displacement values until stable values of stress are achieved within the ROI. The resulting stress values are then converted to force. However, the estimation of force via the constrained traction force microscopy approach is very susceptible to noise because high noise leads to spurious and large force values at the cell boundary. In this regard, although computationally more expensive, unconstrained analysis is preferrable, although sufficient distance between the video frame edges and the cell must exist and determined experimentally.

Parameter calculation and Peak averaging

Once curves of the contractile parameters over time are obtain, an algorithm was implemented to automate the extraction of contractile parameter. For this, the evolution of the parameters over time are calculated from the field of displacement of the beads and stress in each video frame relative to a frame of reference of the cell in its most relaxed state, yielding a curve of the parameters versus time.

As for the bright field contractile analysis, the choice of $frame_{ref}$ is critical for the shape of curves. The selected $frame_{ref}$ is not necessarily the first frame of the video, but instead a frame that showed the cell in its most relaxed state. The selection of a reference $frame_{ref}$ was automated, by first selecting the first frame of the video as initial guess and compared with other frames to select the one that best satisfied the following criteria:

maximize $[m(\mathrm{frame_{ref}}) = \Delta d \approx d_i\left(t_{\mathrm{frame_{ref}}}\right)_{\max} - d_k\left(t_{\mathrm{frame_{ref}}}\right)_{\min}]$, (28)

minimize $[n(\mathrm{frame_{ref}}) = \sum_{i=1}^{N}(d_i\left(t_{\mathrm{frame_{ref}}}\right)\ )]$.(29)

The first criterion ensures a maximal difference between the maximum and minimum points of the displacement curve. However, the solution for this criterion can result in either the desired $\mathrm{frame_{ref}}$ where the cell is in its most relaxed state, or a $\mathrm{frame_{ref}}$ where the cell is in its most contracted state instead. The second criterion selects a $\mathrm{frame_{ref}}$ that minimizes the area under the displacement curve. These two criteria were enough to automatically identify $\mathrm{frame_{ref}}$ as a

frame where the cell is in its most relaxed state. Once frameref was selected, all displacements in regions within the ROI were calculated relative to $frame_{ref}$.

The average displacement of beads in the ROI is defined as:

$d(t_k) = \frac{1}{N}\sum_{k=1}^{N}\sqrt{u_{k,x}^2 + u_{k,y}^2}$, (30)

where k = 1,..., N corresponds to the frame number in the video and (u_x , u_y) is the displacement vector at each pixel.

The average velocity of movement within the ROI was calculated using the first derivative of the mean movement as:

$V(t_k) \approx \frac{\Delta d}{\Delta t} = \frac{d_{k+1} - d_{k-1}}{t_{k+1} - t_{k-1}}$, (3)

where $d_k = d(t_k)$.

The beating rate (br) is defined as the number contractile cycles per unit of time. Two approaches are used to determine br from the curve $d(t_k)$: discrete Fourier transformation of $d(t_k)$ reveal the dominant frequency peaks; or calculation of br as the inverse of period (T) manually defined as the time between adjacent peaks of on the $d(t_k)$ curve in the time domain, calculated as: br $= (\frac{1}{m}\sum_{i=1}^{m} T_i)^{-1}$. Both approaches deliver the same result, although the second approach offers higher flexibility when $d(t_k)$ has high arrhythmicity.

To determine the duration of each contraction, the time between highest contraction velocity and highest relaxation velocity was calculated using each adjacent maximum and minimum of V(t_k) and averaged for all contraction cycles:16

$\hat{t} = \frac{1}{m}\sum_{i=1}^{m}\| t_k|\max_i V(t_k) - t_k|\min_i V(t_k)\|$. (6)

Ideally, the time of each contractile cycle could be calculated from $d(t_k)$ However, this approach is difficult because the exact beginning and end of each contraction curve are often hard to select.

The average total force in the ROI is defined as:

$F(t_k) = \frac{1}{N}\sum_{k=1}^{N}\sqrt{F_{k,x}^2 + F_{k,y}^2}$, (1)

where k = 1,..., N corresponds to the frame number in the video and (F_x , F_y) is the force vector at each pixel.

The zeroth order moment of the traction stress is defined as:

$$\int d^2r\vec{\mathbf{T}}(\vec{\mathbf{r}}) = \tilde{\tilde{\vec{\mathbf{T}}}}(\vec{\mathbf{k}})\Big|_{\vec{\mathbf{k}}=0}$$

where $\mathbf{u}(\vec{\mathbf{r}})$ is displacement vector at $\vec{\mathbf{r}}$, $\vec{\mathbf{T}}(\vec{\mathbf{r}})$ the traction vector at $\underline{\vec{r}}$. This is equal to the net force applied by the cell to the substrate and for isolated adherent cells, this is known a priori to be zero and where $\tilde{\tilde{\vec{\mathbf{T}}}}(0) = 0$ to guarantee no net force results from spurious values.

The shear moment matrix **M** is calculated using approximate derivative of discrete differences at the lowest non-zero wave number Δk_x and Δk_y as:

$$M_{ij} = -(i/2)\int d^2r\left[x_iT_j(\vec{r}) + x_jT_i(\vec{r})\right] = -(i/2)\left[\partial\tilde{T}_j(\vec{k})/\partial k_i + \partial\tilde{T}_i(\vec{k})/\partial k_j\right]\Big|_{\vec{k}=0}$$

$$M_{ij} = \frac{-(i/2)[\tilde{T}_j(\Delta k_i) + \tilde{T}_i(\Delta k_j)]}{|\Delta k|}$$

where M_{xx} and M_{yy} are the total contribution of the cell contraction along the main x and y principal cell axis direction after rotating the $\mathbf{M^{rot}} = \mathbf{R^{-1}MR}$, i.e. $M_{xy}^{rot} = M_{yx}^{rot} = 0$.

The net contractile moment is defined as:

$$\mu = tr(\mathbf{M}) = M_{xx} + M_{yy}$$

The strain energy is defined as:

$$\mathbf{U} = \frac{1}{2}\cdot\int\vec{\boldsymbol{T}}(\vec{\mathbf{r}})\cdot\vec{\mathbf{u}}(\vec{\mathbf{r}})\,dxdy$$

Once these curves are calculated, parameters are calculated by averaging the values for each contractile cycle recorded in the video (on average 5-10 cycles). Contraction cycles are either identified automatically using a peak detection function in Matlab to detect local maximum and local minimum. However, this method often fails to perform optimally when the curves are noisy. Hence, we implemented an algorithm detect the main frequency component in the signal, extracts individual contractile cycle and average these cycles to then extract the average parameters value, their standard deviation and so forth.

The algorithm first uses fast Fourier transform (fft) to filter the signal with a bandpass filter between 0.3 and 10 Hz to remove unwanted low and high frequency noise.

$f(t) = \mathrm{FFT}^{-1}(\mathrm{FFT}(f(t))\cdot\delta)$, where $\delta = \begin{cases} 1, \text{when } 0.3 < \theta_{f(t)} < 10 \\ 0, \text{ when } \theta_{f(t)} < 0.3, or\ \theta_{f(t)} > 10 \end{cases}$, where *f(t)* is the signal as function of time, $\theta_{f(t)}$ is the dominant frequency in the original signal as detected by FFT as explained above.

Then, an autocorrelation is performed on the signal to find the dominant repeating period in the signal and verify that the dominant frequency corresponds to that of the highest signal autocorrelation. The beating rate br and the standard deviation around this beating rate as a measure of arrhythmia is then also calculated:

$$1/\mathrm{br}_{\mathrm{autocorr}(f(t))} = \max\left(\mathrm{findpeak}(f(t) * f(t)\right)$$

Based on this beating rate br and the time position ($t_{n_{\mathrm{peaks}}}$) of each peak of contraction n_{peaks} is identified and each peak is extracted individually.

Then, the most prominent peak is identified and cross-correlated with the original signal to chop the signal at each peak found in the cross-correlation. This allows to ensure the most relevant peaks serves as a basis for the identification of other relevant peaks and that less significant peaks or repeating signal patterns are ignored, such is when the most repeat pattern in the signal is the results of random noise.

$$f_{n_{\mathrm{peak}}}(s) = f\left(\left[t_{n_{\mathrm{peaks}}} - \frac{1}{2\cdot\mathrm{br}_{\mathrm{autocorr}(f(t))}}, t_{n_{\mathrm{peaks}}} + \frac{1}{2\cdot\mathrm{br}_{\mathrm{autocorr}(f(t))}}\right]\right) = f\left(\left[t_{n_{\mathrm{max\ peaks}}} - \frac{1}{2\cdot\mathrm{br}_{\mathrm{autocorr}(f(t))}}, t_{n_{\mathrm{max\,peaks}}} + \frac{1}{2\cdot\mathrm{br}_{\mathrm{autocorr}(f(t))}}\right]\right) * f(t),$$ where s is the time vector of each peak.

Each extracted peak is then aligned around each of their most contracted state, $t_{n_{\mathrm{peaks}}\,\mathrm{max\,contration}}$, and averaged and the standard deviation at each time point is calculated to yield the envelope of the overlapped peaks. The most contracted states are identified and averaged within the peak central region, while the most relaxed level outside the peaks' central region, including their standard deviation along the vertical and time axis for each peak. The peak amplitudes and peak duration at half-height are then calculated. Further, the area under the curve in the contracting, relaxing part and whole of the cycle are integrated as a measure of the total energy of contraction. Finally, the maximum contracting and relaxing speed are computed as describe above. Finally, the strain energy and contractile moments were also computed, and the position of center of force and direction of the contractile moment were calculated as well, as an additional measure of the polarity of the cell.

A benchmarking model for validation and standardization of traction force microscopy analysis tools

Traction force microscopy (TFM) is a well-established tool to measure the biophysical forces exerted by cells on soft substrates. [6,7] It relies on the imaging the displacement of fiducial markers, often fluorescent microspheres embedded in a biocompatible hydrogel of controlled stiffness, to compute the traction stress. In TFM, low image quality or inadequate processing parameters can lead to inaccurate computation of traction stress, especially on stiffer substrates and with noisy images, where bead displacement is small and more challenging to measure. It is therefore crucial to quantify the overall accuracy of the computation, as well as the effect(s) of individual processing parameters and of experimental conditions on the results. Further, to render results from various studies comparable, is important to be able to benchmark the analytical algorithm used.

Here, we developed an *in-silico* model that addresses this need. Our model was developed in Matlab and enables to generate virtual images or video of the contraction of a cell, using as input a single computer-generated or real microscopy image of randomly distributed fluorescent microspheres as well as user-defined cell geometry and contractile stress. This model is built upon Jorge-Peñas et al work.[8] The code is available from our Github page.

Methods

Our model defines a virtual cell and associated force dipole, for which the dimension, orientation, amplitude, area of application, and temporal dynamics can be controlled by design. Using the forward mathematical tensor for the stress-strain relation, the model computes the deformation field that such a force dipole would dynamically induce in a deformable material of the chosen stiffness.[1,6] The calculated deformation field is then imprinted onto the input image through a warping-function image processing to yield a virtual image or video of a hydrogel under dynamic deformation from a cell (**Figure S1**). To investigate the effects of processing parameters on computational output, we used our model to design videos of a virtual ellipsoidal cardiomyocyte (CM) cell of length 100 μm and width 18 μm, at orientations ranging from -90˚ to +90˚. The virtual CM was designed to produce a contraction dipole total force of maximum

amplitude 0.5 μN typical in CMs differentiated from human induced pluripotent stem cells, applying a spatial surface stress distributed as a 2D Gaussian of radius 5 μm at either end of the cell body.

The force amplitude was applied as a time-varying signal following a Gaussian, triangular, or square signal profile. The exact displacement generated by our model dipole was computed with the exact forward mathematical tensor. The displacement fields were used to apply an image warping transformation to the single static image of the fluorescent microspheres in subsequent steps to generate virtual videos of a contracting cell (**Figure S12**).

The displacement field and virtual videos were provided as input to a TFM computation software developed by our lab.[9] Our computational tool makes use of the NCorr algorithm for the calculation of the fluorescent microsphere displacement by image cross-correlation and of Fourier Transform Traction Cytometry for the reconstruction of the traction stress in the Fourier space, as proposed by Butler *et al.* and Sabass *et al.* . [6,7]

Results

The total force recovered was plotted as function of the cell orientation and of the processing parameters for the displacement calculations **Figure S14**.

For our default process parameters, the total force recovered by our TFM analysis algorithm in within an average of 96.8% of the input force when reconstructing traction stress and microsphere displacement from the computer-generated model video, and an average of 93.6% when reconstructing traction stress directly from the exact deformation field computed with the model (Figure S14.a). For a horizontally oriented cell, the force recoveries are 94.9% and 100.2%, respectively. A variation of 5.4% results from changing the orientation of the cell in the image frame. There are two explanations for latter variations. First, digitizing the analysis may induce and propagate digital errors due to pixelization. Second, edge effects are known to impact the calculations of microsphere displacement and force; these effects are more pronounced the closer a cell end is to the edge of the image. In such cases, the numerical noise induced by the edge propagates far enough to be included in the surface integration of the traction stress within the cell boundary, impacting the results. We observe that for a cell that is oriented along the diagonal of the frame, i.e., 45°, the recovered force is within less than 1% of the input, since the distance to the image edges is minimal in this configuration.

Varying the analysis parameters for the DIC step (spacing coefficient and subset radius) generates little difference in terms of average displacement within a cell area, but strongly impacts the reconstruction of the traction force (**Figure S14.b**). In our analytical TFM software, DIC is performed using the open-source Ncorr MATLAB package to track hydrogel deformation by following the position of the embedded fluorescent microspheres. In this algorithm, the image is broken down in sub-windows, defined by the subset radius, and interspaced by a specified spacing coefficient, and the position of each window is identified in subsequent frames via 2D cross-correlation. A spacing coefficient smaller than half the subset radius leads to overlapping windows, in effect oversampling the signal, while a larger spacing coefficient leads to undersampling. The size of each window must be defined empirically as a function of experimental conditions (image noise and resolution, microsphere density, and focal depth). Large window sizes underestimate the local displacement by including too large of an area, while small windows sizes yield noisy results. Finally, the smaller the window size and the larger the spacing between windows, the faster the computation, but often at the expense of accuracy. Under our experimental conditions, a spacing coefficient of 10 pixels and a subset radius of 30 pixels yielded the best compromise between results accuracy and computational performance (**Figure S14.b**). The code is openly accessible here,[10] and this text as a standalone bioRxiv manuscript.[11]

Our model can also serve as a benchmarking tool for determining or optimizing analysis parameters for specific experimental conditions. Indeed, our model can take as input either a virtual image (for example a dark image of dimensions similar to those of the target video, in which bright dots the size of fluorescent microspheres are randomly distributed at controlled density) or a real single image of fluorescent microspheres in the hydrogel, allowing benchmarking against a specific imaging setup.

Overall, our model is a valuable tool to researcher developing TFM algorithms, as it enables validation of the computational accuracy and standardization of results across studies or between laboratories, thereby increasing reproducibility of such assays and increasing the attractiveness of TFM as an approach.

SUPPLEMENTARY FIGURES AND LEGENDS

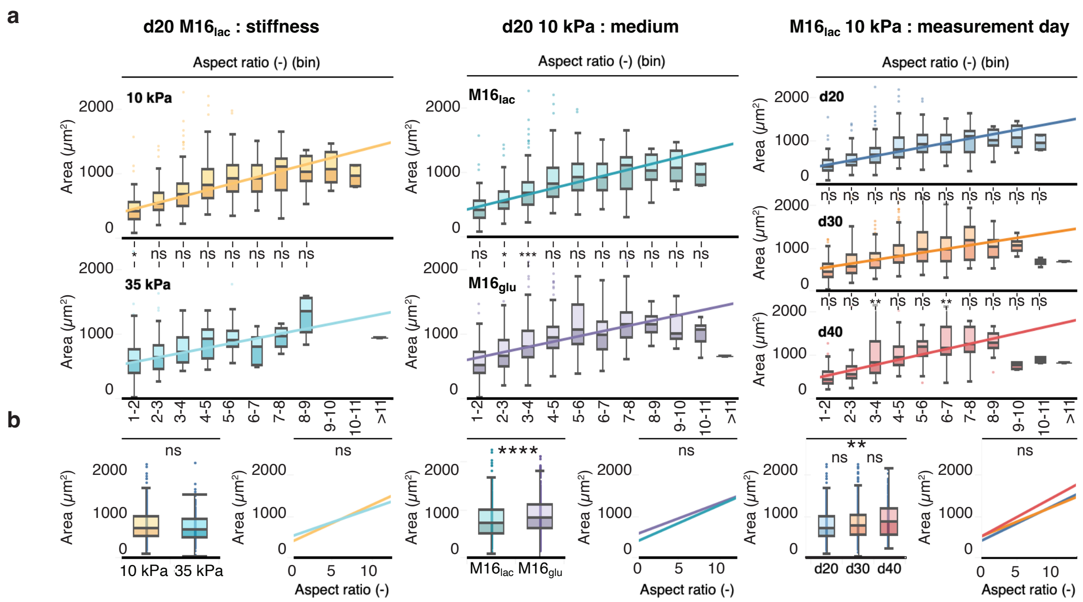

Figure S1: Part 2 of Figure 2: Characterization of cell morphology reveals heterogeneities in populations of hiPSC-CMs. a) The mean spread areas increases as function of aspect ratio (grouped by range). **b)** Overall mean of spread area depends on stiffness, medium, and experimental day, but the slope of spread area versus aspect ratio (comparison of linear regressions) is the same. In all panels, **p* < 0.05, ***p* < 0.005, ****p* < 0.001, *****p* < 0.0001. ns, not significant.

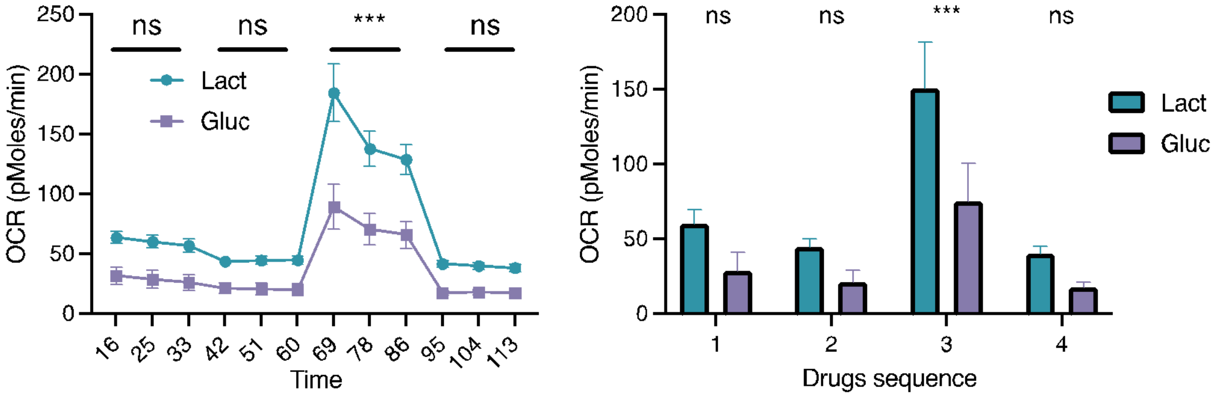

Figure S2: Seahorse metabolic assay for hiPSC-CMs cultured in M16$_{lac}$ and M16$_{glu}$ media. Mean basal and maximal oxygen consumption rate (OCR) is higher for cells in M16$_{lac}$ medium. Statistics with t-test: *$p < 0.05$, **$p < 0.005$, ***$p < 0.001$, ****$p < 0.0001$. ns, not significant, 3 biological replicates, >3 technical replicates, error bar report standard error.

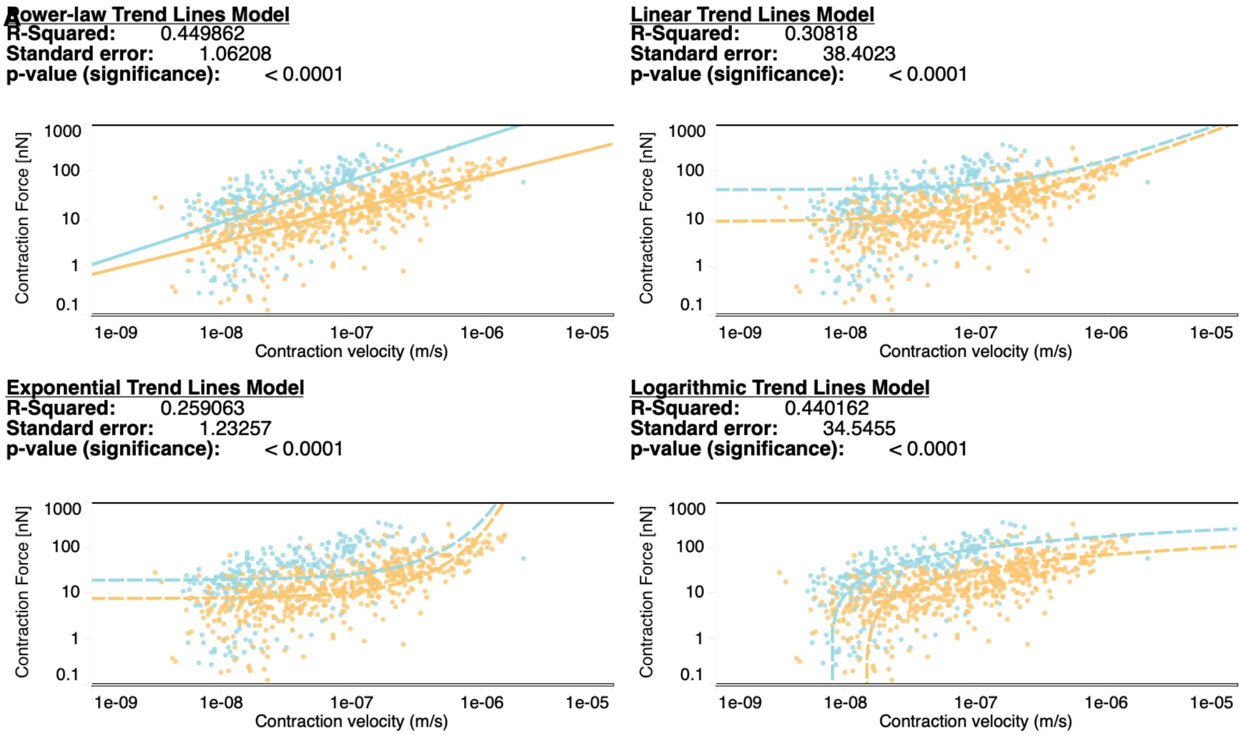

Figure S3: Contraction forces are impacted by substrate stiffness at day 20 in M16$_{lac}$ medium and are best fitted by a power-law relationship.

Power-law Trend Lines Model

A linear trend model was computed for the natural log of Contraction Force [nN] given the natural log of Contraction velocity (m/s). The model is significant at p <= 0.05, meaning that there is a non-zero-slope linear relationship between the natural log of Contraction Force [nN] and the natural log of Contraction velocity. The difference in model fit as a factor Stiffness is significant at p <= 0.05.

Supplementary Table S1: Power law parameters

**Model formula:**	Stiffness*(ln(Contraction velocity (m/s)) + intercept)
**Number of modeled observations:**	837
**Number of filtered observations:**	17
**Model degrees of freedom:**	4
**Residual degrees of freedom (DF):**	833
**Sum squared error (SSE):**	939.632
**Mean squared error (MSE):**	1.12801
**R-Squared:**	0.449862
**Standard error:**	1.06208
**p-value (significance):**	< 0.0001

Analysis of Variance:

A one-sided F test was used for this comparison with the following parameters:

Field	DF	SSE	MSE	F	p-value
**Stiffness**	2	258.19928	129.1	114.449	< 0.0001

Supplementary Table S2: Individual trend lines:

A one-sided T-test was used against the null hypothesis of a zero slope and for the statistical comparison of models with the following parameters:

Stiffness	p-value	DF	Term	Value	SE	t-value	p-value
35kPa	< 0.0001	284	ln(Contraction velocity (m/s))	0.891683	0.0686588	12.9872	< 0.0001
			intercept	18.5952	1.19143	15.6074	< 0.0001
10kPa	< 0.0001	549	ln(Contraction velocity (m/s))	0.686513	0.0308868	22.2267	< 0.0001
			intercept	13.8682	0.507475	27.3279	< 0.0001

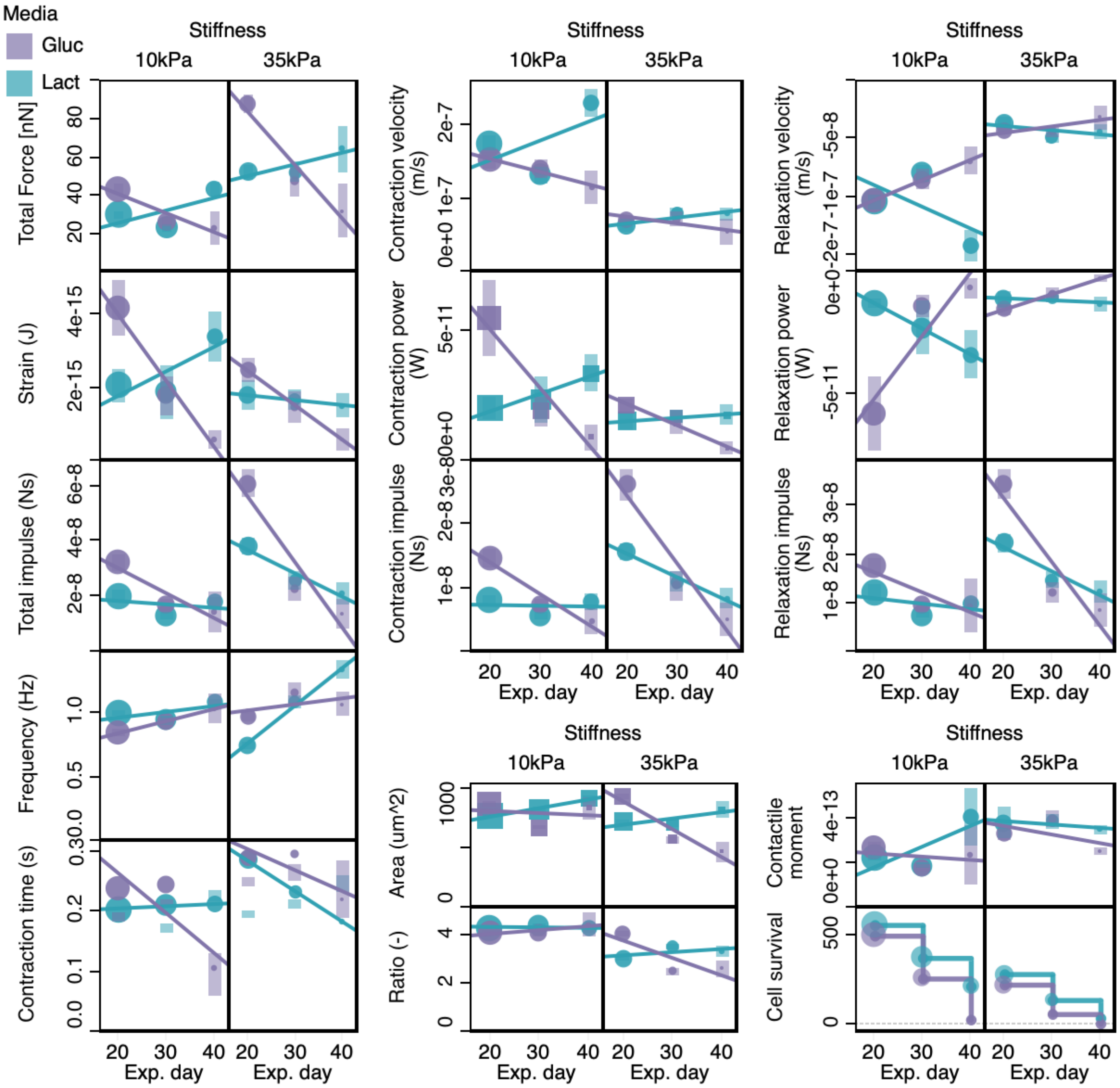

Figure S4 Evolution of all the measured contractile parameters over the time course experiment for both substrate stiffness and medium compositions. (see **Table S3** for data)

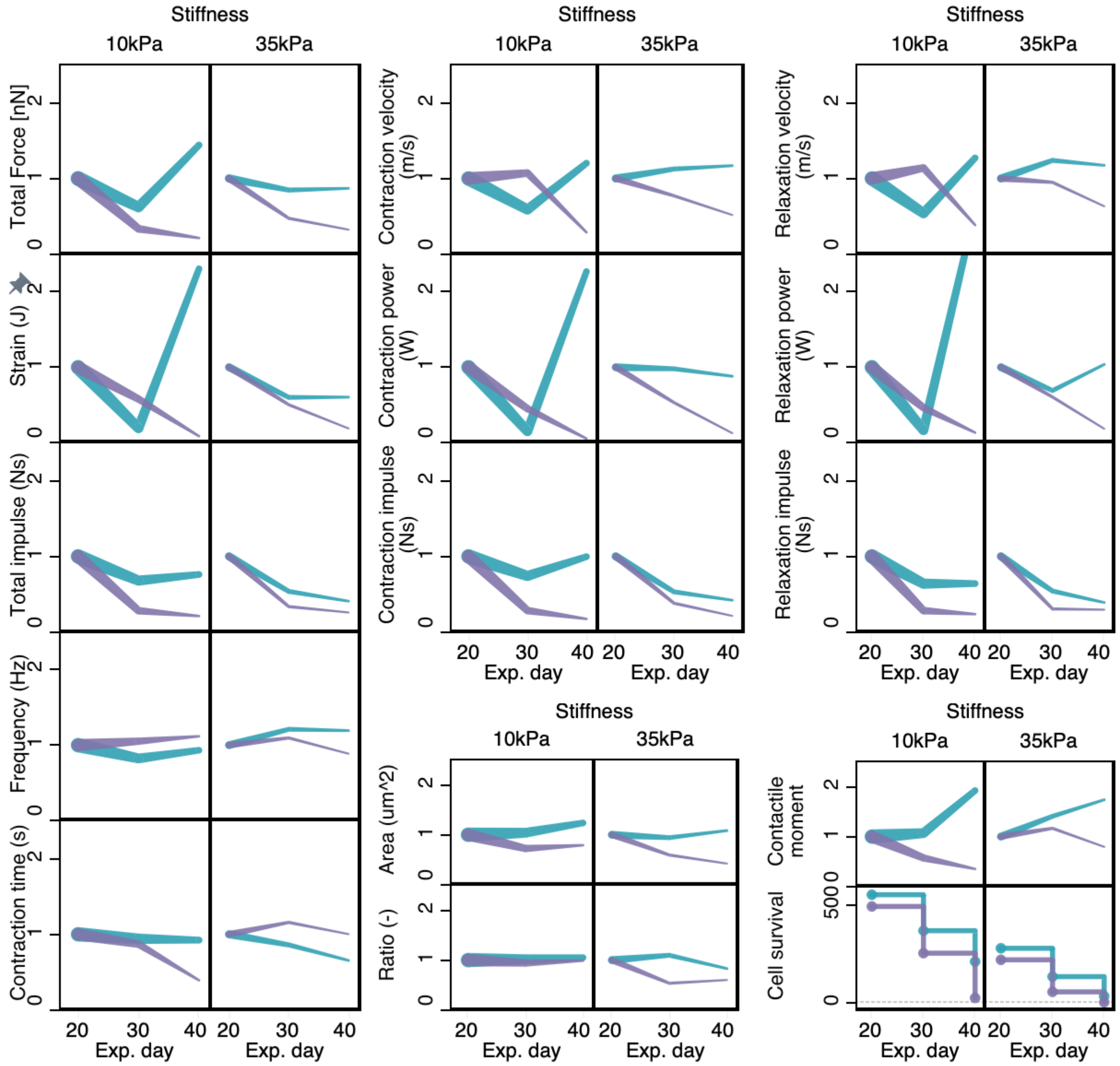

Figure S5 Day 20-normalized evolution of all the measured contractile parameters over the time course experiment for both substrate stiffness and medium compositions.

mean value<br>standard deviation<br>n	Media / Stiffness / Exp. day											
	M16-1						M16-2					
	10kPa			35kPa			10kPa			35kPa		
	d20	d30	d40	d20	d30	d40	d20	d30	d40	d20	d30	d40
Avg. Area (um^2)	7.64e+02	8.27e+02	9.11e+02	7.16e+02	7.02e+02	8.23e+02	8.72e+02	6.73e+02	8.35e+02	9.37e+02	5.69e+02	4.64e+02
	3.50e+02	4.23e+02	4.60e+02	2.91e+02	3.74e+02	4.36e+02	3.70e+02	3.15e+02	4.88e+02	3.82e+02	2.60e+02	2.19e+02
	591	377	220	282	138	45	498	259	29	224	59	7
Avg. Aspect ratio (-)	4.27e+00	4.38e+00	4.23e+00	3.02e+00	3.49e+00	3.29e+00	4.09e+00	4.03e+00	4.41e+00	4.03e+00	2.47e+00	2.58e+00
	1.95e+00	2.05e+00	2.22e+00	1.83e+00	1.69e+00	1.51e+00	2.00e+00	1.84e+00	2.63e+00	1.84e+00	1.23e+00	9.10e-01
	591	377	220	282	138	45	498	259	29	224	59	7
Avg. Average contraction displacement (m)	2.09e-08	1.49e-08	2.89e-08	7.51e-09	8.61e-09	7.77e-09	1.94e-08	1.73e-08	1.27e-08	9.33e-09	9.27e-09	5.48e-09
	3.51e-08	2.69e-08	3.94e-08	1.77e-08	1.08e-08	7.07e-09	3.36e-08	2.86e-08	1.36e-08	8.41e-09	8.74e-09	4.24e-09
	566	375	218	282	138	40	494	257	28	223	59	6
Avg. Contraction Force [nN]	3.02e+01	2.33e+01	4.33e+01	5.21e+01	5.21e+01	6.42e+01	4.30e+01	2.64e+01	2.32e+01	8.75e+01	4.73e+01	3.21e+01
	4.71e+01	3.55e+01	5.64e+01	6.26e+01	6.24e+01	7.42e+01	6.09e+01	4.09e+01	4.57e+01	7.57e+01	5.77e+01	3.39e+01
	556	372	214	282	137	37	496	257	28	223	59	6
Avg. Contraction impulse (Ns)	8.01e-09	5.52e-09	7.76e-09	1.56e-08	1.09e-08	8.23e-09	1.47e-08	7.38e-09	4.58e-09	2.62e-08	1.03e-08	4.92e-09
	1.90e-08	1.17e-08	1.83e-08	2.35e-08	1.82e-08	9.49e-09	3.11e-08	1.31e-08	1.09e-08	3.63e-08	1.76e-08	6.25e-09
	555	370	214	282	137	36	487	255	28	223	59	6
Avg. Contraction power (W)	1.99e-11	2.33e-11	3.37e-11	1.47e-11	1.66e-11	1.74e-11	5.48e-11	1.88e-11	8.85e-12	2.12e-11	1.44e-11	4.63e-12
	1.03e-10	1.68e-10	1.05e-10	5.30e-11	2.85e-11	2.52e-11	3.31e-10	9.31e-11	2.66e-11	3.73e-11	3.55e-11	6.18e-12
	588	374	218	281	137	45	493	259	29	221	58	7
Avg. Contraction time (s)	2.03e-01	2.11e-01	2.11e-01	2.86e-01	2.31e-01	1.84e-01	2.40e-01	2.44e-01	1.06e-01	2.91e-01	2.94e-01	2.20e-01
	1.90e-01	2.18e-01	1.37e-01	2.15e-01	1.58e-01	5.44e-02	2.05e-01	1.37e-01	1.62e-01	1.97e-01	2.42e-01	1.01e-01
	530	361	210	262	134	40	445	235	27	206	57	6
Avg. Contraction velocity (m/s)	1.75e-07	1.32e-07	2.30e-07	6.16e-08	7.89e-08	7.78e-08	1.51e-07	1.39e-07	1.15e-07	7.11e-08	7.09e-08	5.28e-08
	2.64e-07	2.24e-07	2.93e-07	1.50e-07	9.01e-08	6.02e-08	2.42e-07	2.14e-07	1.23e-07	6.76e-08	7.16e-08	4.24e-08
	566	375	218	282	138	40	494	257	28	223	59	6
Avg. Frequency (autocorr. of peaks) (Hz)	9.96e-01	9.42e-01	1.09e+00	7.48e-01	1.09e+00	1.34e+00	8.45e-01	9.28e-01	1.04e+00	9.77e-01	1.15e+00	1.07e+00
	5.57e-01	6.13e-01	4.22e-01	4.69e-01	6.03e-01	5.20e-01	5.68e-01	6.00e-01	6.44e-01	5.79e-01	7.02e-01	2.64e-01
	591	376	220	282	138	45	498	259	29	224	59	7
Avg. Lambda	4.71e-10	5.40e-10	4.78e-10	1.89e-10	9.18e-11	2.58e-10	4.93e-10	3.20e-10	2.56e-10	7.58e-11	1.52e-10	1.28e-16
	1.32e-09	1.43e-09	1.22e-09	4.77e-10	3.24e-10	5.56e-10	1.37e-09	1.05e-09	1.01e-09	2.97e-10	4.32e-10	0.00e+00
	591	377	220	282	138	45	498	259	29	224	59	7
Avg. Length (um)	6.40e+01	6.69e+01	6.92e+01	5.14e+01	5.51e+01	5.78e+01	6.65e+01	5.82e+01	6.73e+01	6.92e+01	4.20e+01	3.93e+01
	2.55e+01	2.70e+01	3.15e+01	2.23e+01	2.42e+01	2.44e+01	2.56e+01	2.26e+01	3.00e+01	2.71e+01	1.79e+01	1.58e+01
	591	377	220	282	138	45	498	259	29	224	59	7
Avg. Relaxation impulse (Ns)	1.20e-08	7.33e-09	9.78e-09	2.21e-08	1.46e-08	1.23e-08	1.74e-08	9.40e-09	9.28e-09	3.44e-08	1.20e-08	8.26e-09
	3.71e-08	1.09e-08	1.27e-08	3.12e-08	2.05e-08	1.46e-08	3.37e-08	1.58e-08	2.89e-08	4.45e-08	1.67e-08	8.43e-09
	556	372	214	282	137	37	496	257	28	223	59	6
Avg. Relaxation power (W)	-1.34e-11	-2.35e-11	-3.43e-11	-1.16e-11	-1.14e-11	-1.33e-11	-5.85e-11	-1.44e-11	-6.96e-12	-1.59e-11	-9.78e-12	-3.24e-12
	4.48e-11	2.13e-10	1.48e-10	5.77e-11	2.05e-11	2.04e-11	3.39e-10	6.91e-11	2.24e-11	3.71e-11	2.42e-11	4.34e-12
	588	374	218	281	137	45	495	259	29	221	58	7
Avg. Relaxation velocity (m/s)	-1.05e-07	-8.00e-08	-1.42e-07	-3.66e-08	-4.93e-08	-4.39e-08	-1.04e-07	-8.51e-08	-6.92e-08	-4.33e-08	-4.45e-08	-3.22e-08
	1.58e-07	1.38e-07	2.06e-07	8.67e-08	5.87e-08	3.95e-08	1.85e-07	1.44e-07	6.69e-08	4.37e-08	3.94e-08	2.44e-08
	566	375	218	282	138	40	494	257	28	223	59	6
Avg. Strain (J)	2.03e-15	1.85e-15	3.37e-15	1.76e-15	1.65e-15	1.49e-15	4.15e-15	1.77e-15	5.38e-16	2.45e-15	1.46e-15	5.62e-16
	1.10e-14	1.44e-14	9.82e-15	6.85e-15	3.46e-15	2.10e-15	1.71e-14	8.53e-15	1.32e-15	5.05e-15	3.28e-15	7.23e-16
	577	376	219	281	138	40	495	258	28	222	59	5
Avg. Total force tot (N)	3.02e-08	2.33e-08	4.33e-08	5.21e-08	5.21e-08	6.42e-08	4.30e-08	2.64e-08	2.32e-08	8.75e-08	4.73e-08	3.21e-08
	4.71e-08	3.55e-08	5.64e-08	6.26e-08	6.24e-08	7.42e-08	6.09e-08	4.09e-08	4.57e-08	7.57e-08	5.77e-08	3.39e-08
	556	372	214	282	137	37	496	257	28	223	59	6
Avg. Total force x (N)	2.58e-08	2.04e-08	3.77e-08	4.22e-08	4.53e-08	5.31e-08	3.42e-08	2.18e-08	1.87e-08	7.34e-08	4.21e-08	2.84e-08
	4.02e-08	3.24e-08	4.94e-08	5.01e-08	5.49e-08	6.20e-08	4.96e-08	3.66e-08	3.23e-08	6.54e-08	5.18e-08	2.94e-08
	562	373	218	282	137	38	495	257	28	224	59	6
Avg. Total force y (N)	1.16e-08	8.99e-09	1.46e-08	2.61e-08	2.24e-08	2.61e-08	1.99e-08	1.13e-08	1.13e-08	3.97e-08	1.87e-08	1.38e-08
	2.22e-08	1.12e-08	2.23e-08	3.64e-08	2.81e-08	3.06e-08	2.99e-08	1.37e-08	2.67e-08	3.32e-08	1.97e-08	1.17e-08
	573	376	214	282	138	40	495	258	28	222	59	6
Avg. Total impulse (Ns)	2.00e-08	1.29e-08	1.75e-08	3.77e-08	2.56e-08	2.08e-08	3.19e-08	1.67e-08	1.39e-08	6.06e-08	2.23e-08	1.32e-08
	5.42e-08	2.15e-08	2.97e-08	5.23e-08	3.63e-08	2.35e-08	6.15e-08	2.75e-08	3.96e-08	7.74e-08	3.25e-08	1.34e-08
	555	370	214	282	137	36	487	255	28	223	59	6
Avg. Width (um)	1.56e+01	1.62e+01	1.74e+01	1.88e+01	1.65e+01	1.83e+01	1.73e+01	1.53e+01	1.69e+01	1.80e+01	1.77e+01	1.52e+01
	3.48e+00	4.25e+00	4.20e+00	4.89e+00	4.03e+00	4.44e+00	4.17e+00	3.88e+00	5.73e+00	3.55e+00	3.37e+00	1.98e+00
	591	377	220	282	138	45	498	259	29	224	59	7

Table S3: Summary statistics of longitudinal study on 10 and 35 kPa substrate stiffnesses and in $M16_{lac}$ and $M16_{gluc}$ media.

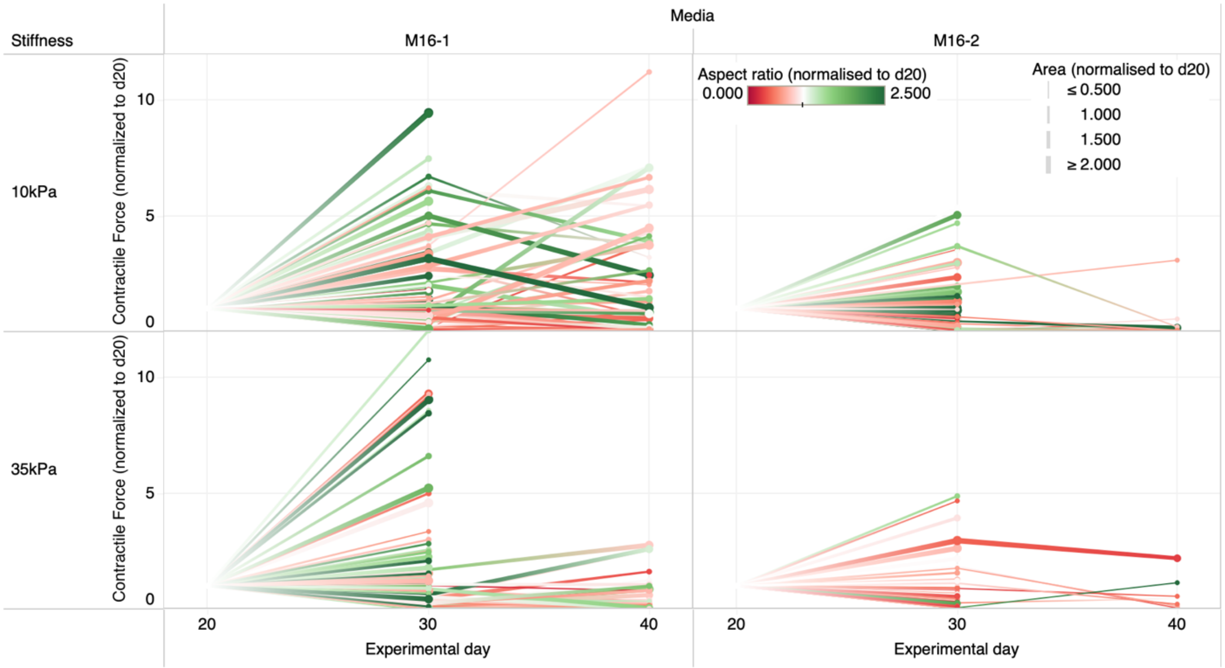

Figure S6 Evolution of the measured contractile force over the time course experiment for both substrate stiffness and medium compositions, showing how higher stiffness and M16gluc medium result in reduced maturation and accelerated cell death.

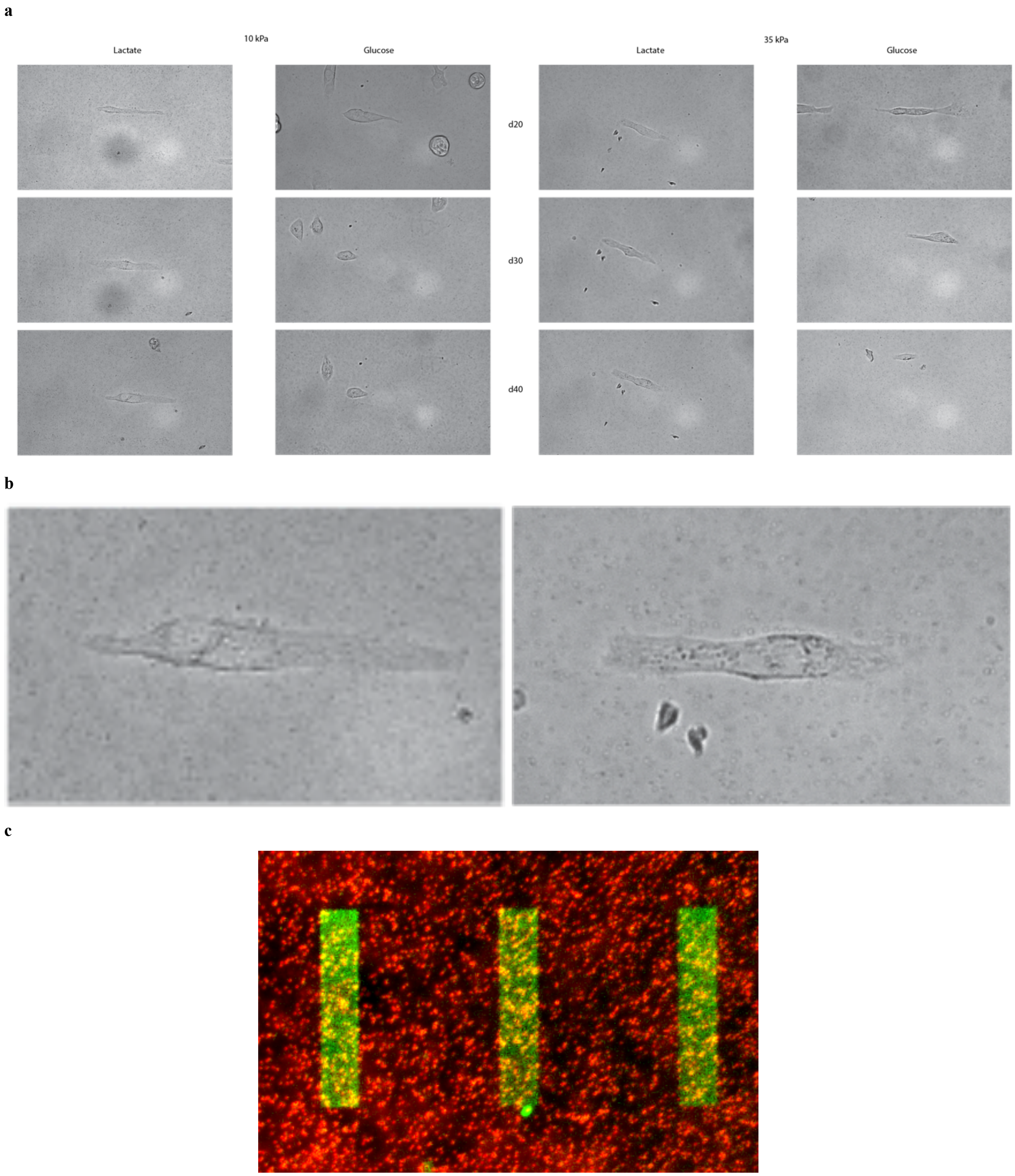

Figure S7 Representative images of single hiPSC-CMs during timelapse imaging. a) Three time points, two media and two substrate stiffness conditions. b) Close of hiPSC-CMs in M16-1 medium at d40 showing the elongated shape provided by the adhesion cues on the micropattern hydrogel substrates. c) Representative micrograph of micropatterns on the hydrogel substrate, with GFP-labelled fibronectin protein spiked into Matrigel and GFP-fluorescent polystyrene microspheres embedded in the polyacrylamide hydrogel.

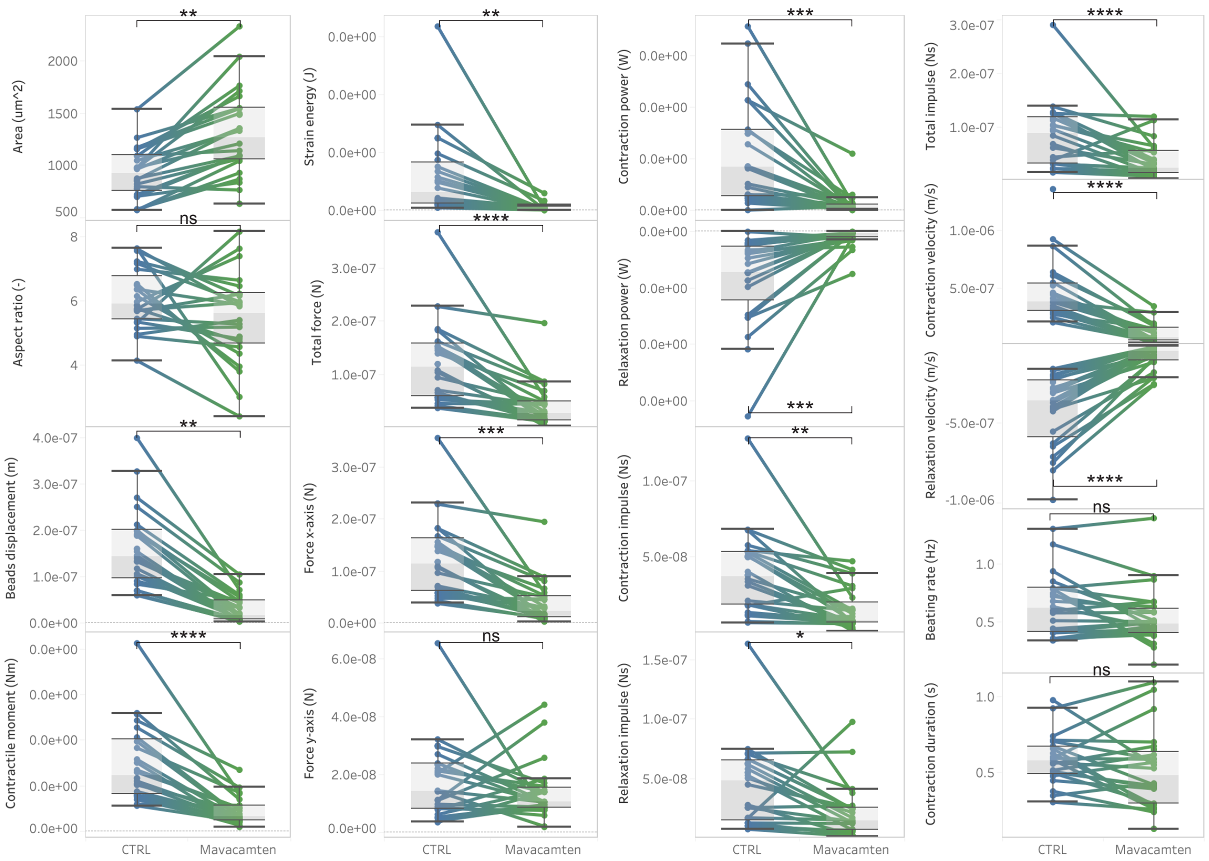

Figure S8: Mavacamten treatment on 10 kPa substrate stiffness. Statistics with t-test: *$p < 0.05$, **$p < 0.005$, ***$p < 0.001$, ****$p < 0.0001$. ns, not significant. (see Table S4 for the statistics)

Mean value<br>Standard deviation<br>Number of sample	CTRL vs MAVA<br>Control		Mavacamten
Area (um^2)	9.28e+02<br>2.39e+02<br>24	0.0023	1.31e+03<br>4.12e+02<br>24
Aspect ratio (-)	6.09e+00<br>9.34e-01<br>24	0.2559	5.46e+00<br>1.38e+00<br>24
Beads displacement (m)	1.60e-07<br>8.59e-08<br>24	0.0023	2.90e-08<br>2.95e-08<br>24
Contactile moment (Nm)	1.50e-12<br>8.56e-13<br>24	< 0.0001	4.38e-13<br>3.08e-13<br>24
Contraction duration (s)	5.84e-01<br>1.60e-01<br>24	0.4350	5.16e-01<br>2.52e-01<br>24
Contraction impulse (Ns)	3.92e-08<br>2.69e-08<br>24	0.0023	1.51e-08<br>1.31e-08<br>24
Contraction power (W)	1.07e-10<br>1.01e-10<br>24	0.0002	1.08e-11<br>2.24e-11<br>24
Contraction velocity (m/s)	4.66e-07<br>2.66e-07<br>24	< 0.0001	9.45e-08<br>8.50e-08<br>24
Force x-axis (N)	1.25e-07<br>7.58e-08<br>24	0.0001	3.73e-08<br>4.17e-08<br>24
Force y-axis (N)	1.78e-08<br>1.36e-08<br>24	0.4350	1.37e-08<br>9.97e-09<br>24
Frequency (Hz)	6.39e-01<br>2.64e-01<br>24	0.4350	5.36e-01<br>2.52e-01<br>24
Relaxation impulse (Ns)	4.46e-08<br>3.49e-08<br>24	0.0409	2.11e-08<br>2.29e-08<br>24
Relaxation power (W)	-8.56e-11<br>7.68e-11<br>24	0.0002	-9.70e-12<br>1.61e-11<br>24
Relaxation velocity (m/s)	-4.21e-07<br>2.24e-07<br>24	< 0.0001	-8.80e-08<br>8.04e-08<br>24
Strain energy (J)	2.87e-14<br>3.53e-14<br>24	0.0037	2.09e-15<br>3.28e-15<br>24
Total force (N)	1.25e-07<br>7.80e-08<br>24	< 0.0001	3.91e-08<br>4.11e-08<br>24
Total impulse (Ns)	8.38e-08<br>6.09e-08<br>24	< 0.0001	3.62e-08<br>3.24e-08<br>24

Table S4: Summary statistics of Mavacamten treatment on 10 kPa substrate stiffness. Data were compared with multiple parametric t-test with 95% confidence interval corrected for multiple comparison using Holm-Sidak method: *$p < 0.05$, **$p < 0.005$, ***$p < 0.001$, ****$p < 0.0001$. ns, not significant.

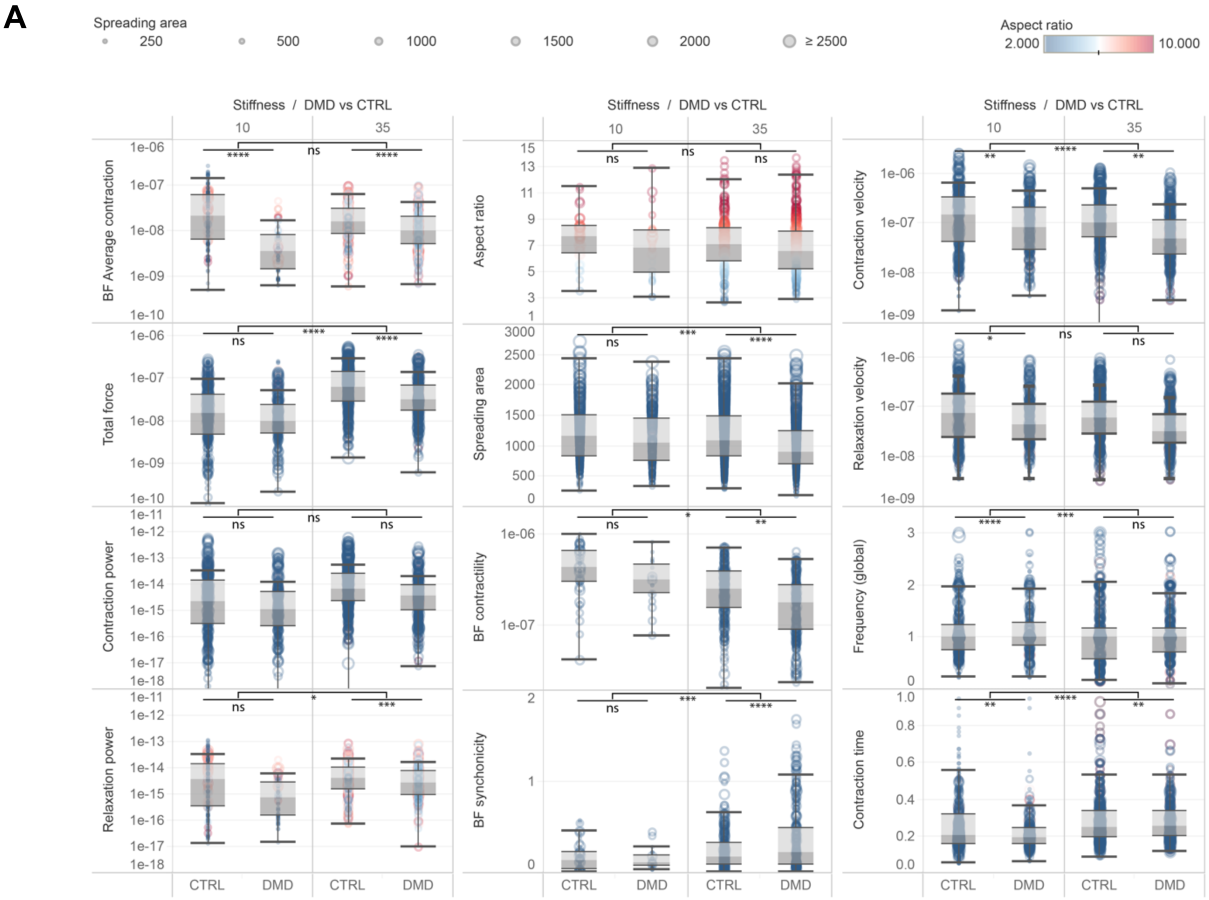

Figure S9: Multiple parameters measured on cardiomyocytes or without DMD mutations on 10 kPa and 35 kPa substrates. DMD hiPSC-CMs clearly demonstrate a general contractile deficiency that is exacerbated on stiffer fibrotic-like substrates. Statistics with t-test: **p* < 0.05, ***p* < 0.005, ****p* < 0.001, *****p* < 0.0001. ns, not significant. (see Table S5 for the statistics)

Mean value<br>Standard deviation<br>Number of sample	DMD vs CTRL / Stiffness<br>CTRL<br>10 kPa		35 kPa		DMD<br>10 kPa		35 kPa
**Aspect ratio**	7.56e+00<br>1.80e+00<br>35	0.6296	7.15e+00<br>2.14e+00<br>177	0.5622	6.87e+00<br>2.45e+00<br>24	0.6843	6.92e+00<br>2.22e+00<br>282
**Beads diplacement**	4.28e-08<br>5.03e-08<br>154	< 0.0001	3.13e-08<br>4.14e-08<br>233	< 0.0001	6.47e-09<br>8.09e-09<br>61	< 0.0001	1.63e-08<br>1.72e-08<br>282
**Brightfield contractility**	4.87e-07<br>2.50e-07<br>55	0.7502	3.49e-07<br>7.55e-07<br>251	0.0499	3.50e-07<br>1.92e-07<br>19	0.0083	2.02e-07<br>1.33e-07<br>244
**Brightfield synchonicity**	1.70e-01<br>1.55e-01<br>55	0.9908	2.42e-01<br>2.17e-01<br>256	0.2091	1.48e-01<br>1.16e-01<br>21	< 0.0001	3.59e-01<br>3.58e-01<br>254
**Contraction power**	7.99e-14<br>1.17e-12<br>492	0.3574	3.50e-14<br>1.16e-13<br>735	0.0994	1.24e-14<br>4.76e-14<br>295	0.9477	1.88e-14<br>1.20e-13<br>692
**Contraction time**	2.49e-01<br>1.43e-01<br>490	0.03463	3.04e-01<br>1.76e-01<br>728	< 0.0001	2.15e-01<br>1.19e-01<br>289	0.02833	2.76e-01<br>1.10e-01<br>601
**Contraction velocity**	3.10e-07<br>9.19e-07<br>517	0.0012	1.88e-07<br>2.23e-07<br>730	< 0.0001	1.81e-07<br>2.65e-07<br>295	0.0052	1.04e-07<br>1.63e-07<br>692
**Force x-axis**	3.69e-08<br>4.72e-08<br>142	0.9717	1.27e-07<br>2.40e-07<br>194	0.0017	2.69e-08<br>3.10e-08<br>50	< 0.0001	5.29e-08<br>4.82e-08<br>238
**Force y-axis**	3.55e-08<br>4.11e-08<br>135	0.9907	9.69e-08<br>1.42e-07<br>186	0.0027	3.12e-08<br>4.12e-08<br>49	< 0.0001	5.03e-08<br>4.65e-08<br>234
**Frequency**	1.03e+00<br>4.72e-01<br>518	< 0.0001	9.88e-01<br>5.80e-01<br>727	< 0.0001	1.19e+00<br>5.46e-01<br>290	0.1217	1.05e+00<br>4.92e-01<br>601
**Major Axis Length**	1.05e+02<br>2.27e+01<br>35	0.0061	9.58e+01<br>2.65e+01<br>177	< 0.0001	8.29e+01<br>2.69e+01<br>24	0.0546	8.97e+01<br>2.45e+01<br>282
**Minor Axis Length**	1.41e+01<br>2.42e+00<br>35	0.0573	1.37e+01<br>2.50e+00<br>177	0.0039	1.25e+01<br>2.58e+00<br>24	0.4193	1.33e+01<br>2.44e+00<br>282
**Relaxation power**	-1.24e-14<br>2.10e-14<br>154	0.7803	-3.33e-14<br>1.22e-13<br>234	0.0030	-2.30e-15<br>3.84e-15<br>61	0.0001	-6.72e-15<br>1.07e-14<br>282
**Relaxation velocity**	-1.59e-07<br>2.67e-07<br>490	0.0234	-1.19e-07<br>1.57e-07<br>730	0.0876	-1.12e-07<br>1.83e-07<br>295	0.3167	-6.69e-08<br>1.14e-07<br>690
**Spreading area**	1.20e+03<br>4.68e+02<br>294	0.0922	1.16e+03<br>4.64e+02<br>635	< 0.0001	1.11e+03<br>4.52e+02<br>223	< 0.0001	9.81e+02<br>4.06e+02<br>660
**Time between main peaks**	-1.24e-14<br>2.10e-14<br>154	0.7803	-3.33e-14<br>1.22e-13<br>234	0.0030	-2.30e-15<br>3.84e-15<br>61	0.0001	-6.72e-15<br>1.07e-14<br>282
**Total force**	3.96e-08<br>8.23e-08<br>489	0.6012	1.36e-07<br>2.18e-07<br>729	< 0.0001	2.66e-08<br>4.26e-08<br>294	< 0.0001	6.07e-08<br>9.44e-08<br>686

Table S5: Summary statistics of DMD vs control on 10 kPa and 35 kPa substrate stiffness. data were compared with multiple parametric t-test with 95% confidence interval corrected for multiple comparison using Holm-Sidak method: *$p < 0.05$, **$p < 0.005$, ***$p < 0.001$, ****$p < 0.0001$. ns, not significant.

Classification	hiPSC	lines	Gene	Age	Tissue	Gender	Source	Used in study
DCM	DMD#1	DMD	(c.3638_3650del)	6	PBMC	male	Stanford	DMD study
DCM	DMD#16	DMD	(c.10171C>T)	10	dermal fibroblasts	male	University of Nottingham	DMD study
DCM	DMD#19	DMD	(c.4918_4919delACinsTG)	8	dermal fibroblasts	male	University of Nottingham	DMD study
DCM	DMD#2	DMD	(c.6599 C>G)	12	PBMC	male	Stanford	DMD study
DCM	DMD#3	DMD	(c.9204_9207del)	9	PBMC	male	Stanford	DMD study
DCM	UC1015-6	DMD isogenic	Exon 1: c.263del of UC3.4	unreported	Urine	male	University of Washington	DMD study
Healthy	DMD16iso	Isogenic control	control of DMD #16	10	dermal fibroblasts	male	University of Nottingham	DMD study
Healthy	DMD19iso	Isogenic control	control of DMD #19	8	dermal fibroblasts	male	University of Nottingham	DMD study
Healthy	JG control	Healthy	control	45	unerported	male	Stanford	DMD study
Healthy	Norm1	Healthy	control	62	unerported	male	Harvard	DMD study
Healthy	Norm2	Healthy	control	48	unerported	female	Harvard	DMD study
Healthy	UC3-4	Healthy	control	unreported	Urine	male	University of Washington	DMD study
Healthy	WTC	Healthy	control	30	Skin fibroblasts	male - Japan	GM25256iPSC Allen Institute-Coriel	Time-course study & Mavacamten study

Table S6: Summary of cell lines used in the various studies.

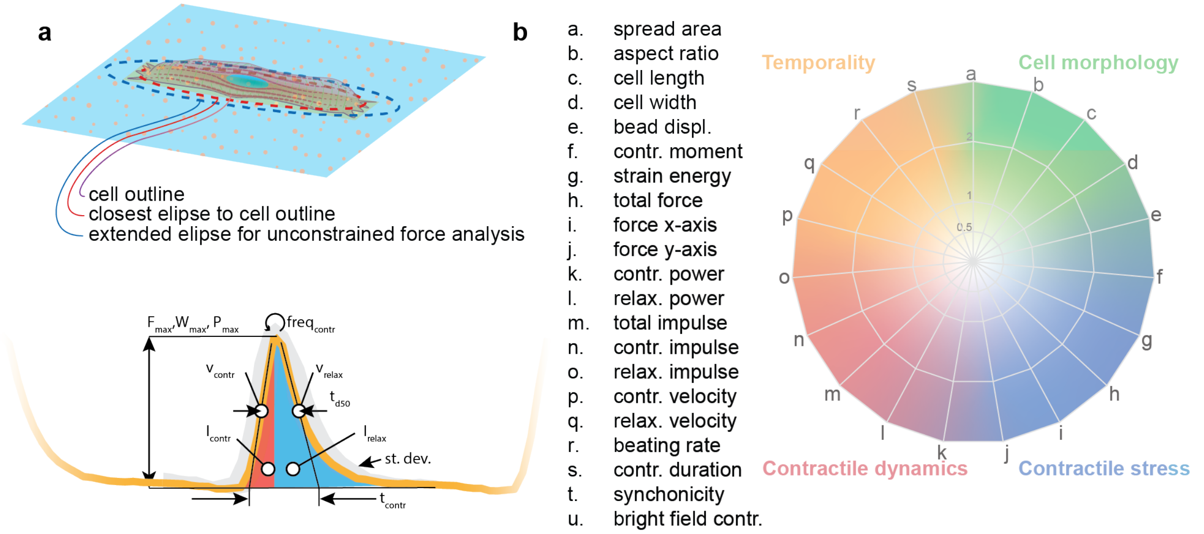

Figure S10: Graphical description of traction force parameter extraction and spider plots. A) Description of cell area and ellipse fitting and scaling used for constrained traction force analysis; description of contraction peak analysis. After automated extraction of multiple contraction cycle peaks, peaks are aligned and averaged to calculate the envelope and relevant peak parameters: d_{max} = beads displacement, F_{max} = max force, W_{max} = max strain energy, P_{max} = max power, v_{contr} = contraction velocity, v_{relax} = relaxation velocity, t_{contr} = contraction duration, t_{50} = contraction duration at half-height, I_{contr} = contraction impulse (area under the rising part of the curve), I_{contr} = relaxation impulse (area under the receding part of the curve), $freq_{contr}$ = contraction frequency (beating rate), st. dev. = standard deviation envelope. **B)** The spider plots background color regroups related parameters to: cell morphology, contractile stress, contractile dynamics, temporality of contraction.

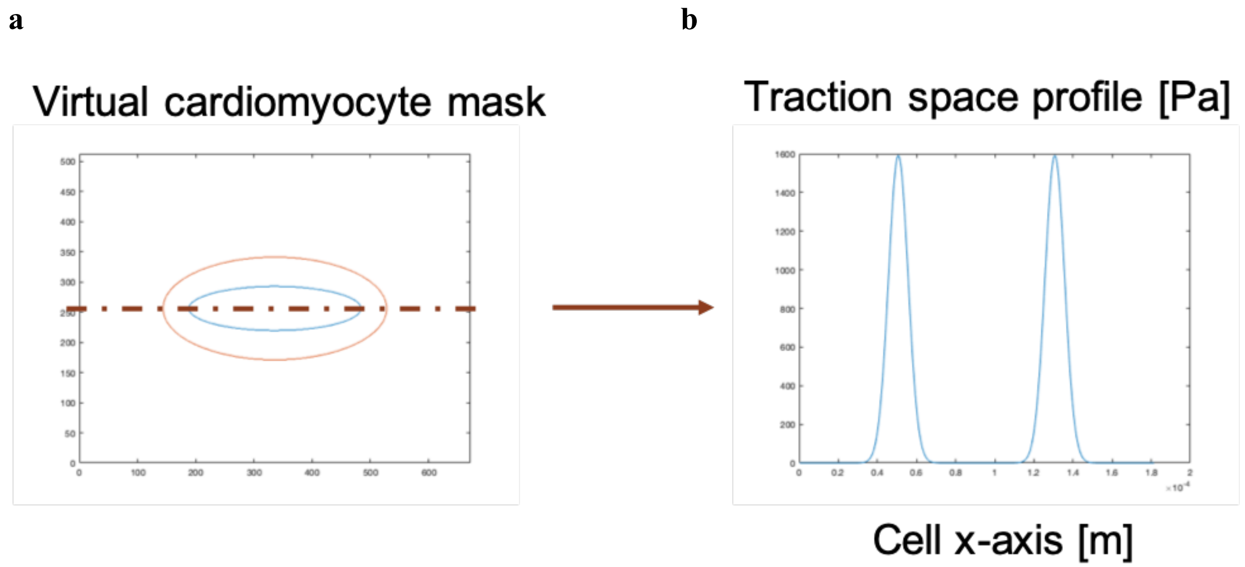

Figure S11: A virtual cell is defined as a ellipse of elongated aspect ratio (a) that produces a contracting force dipole (b).

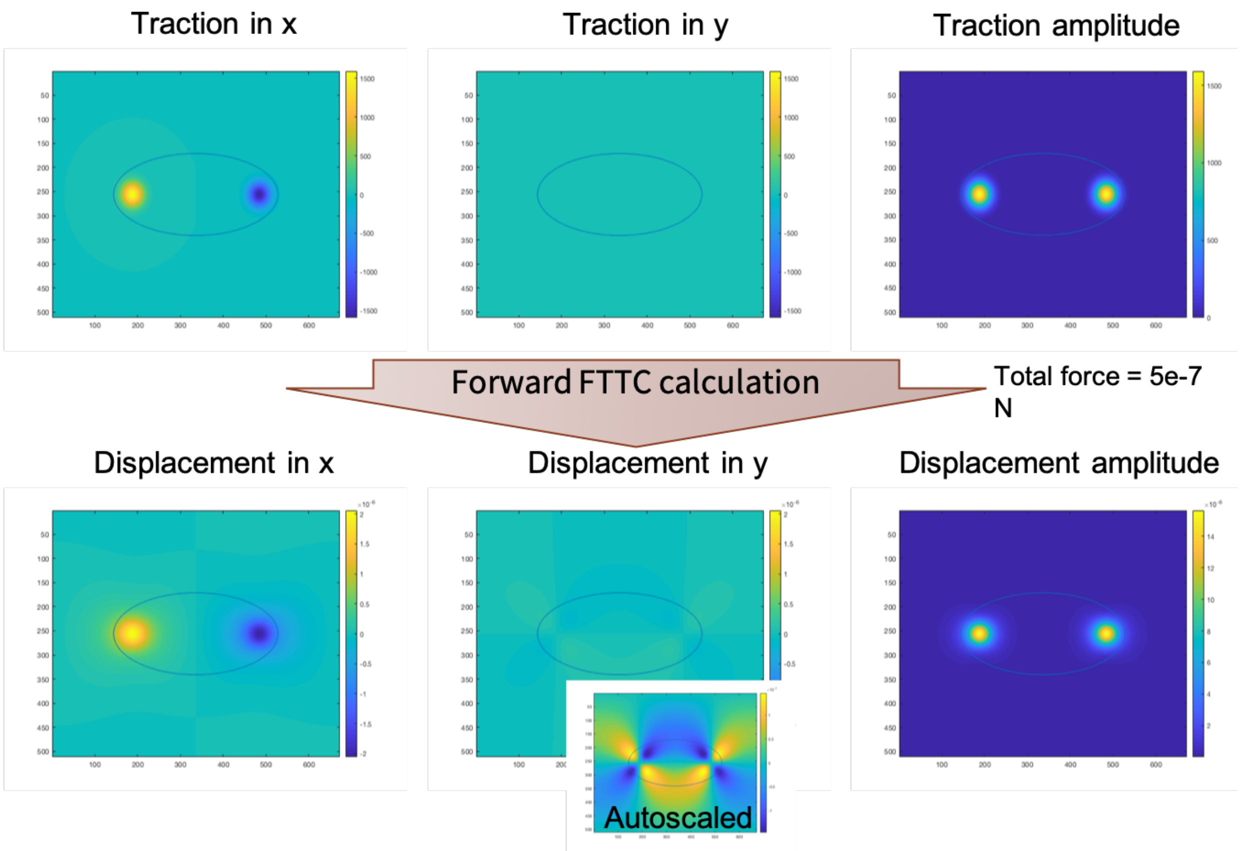

Figure S12: The traction force dipole (Top) is used as input in the model to compute the exact hydrogel deformation displacement (Bottom). Inset in y displacement shows the displacement in the y-direction with tight color scale. This calculated displacement is used to deform the static image of the fluorescent microsphere**.**

a

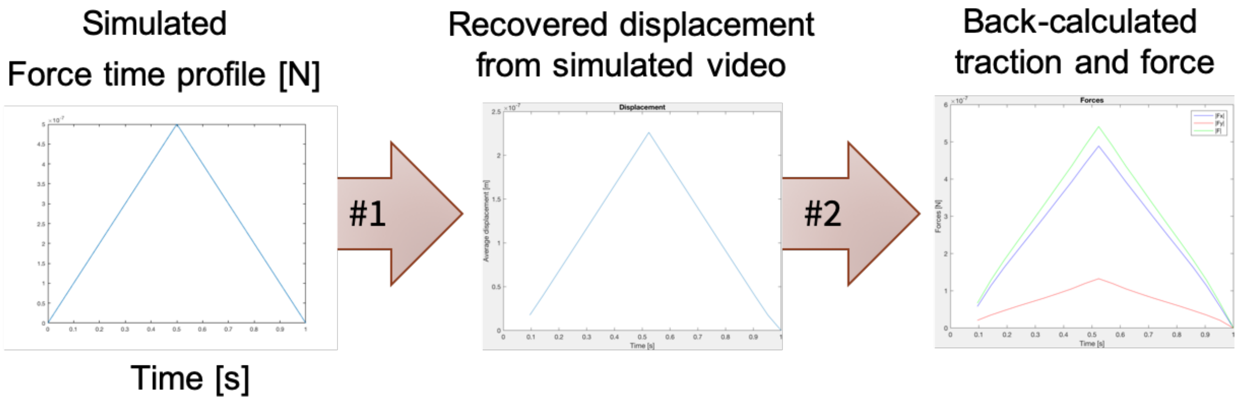

b

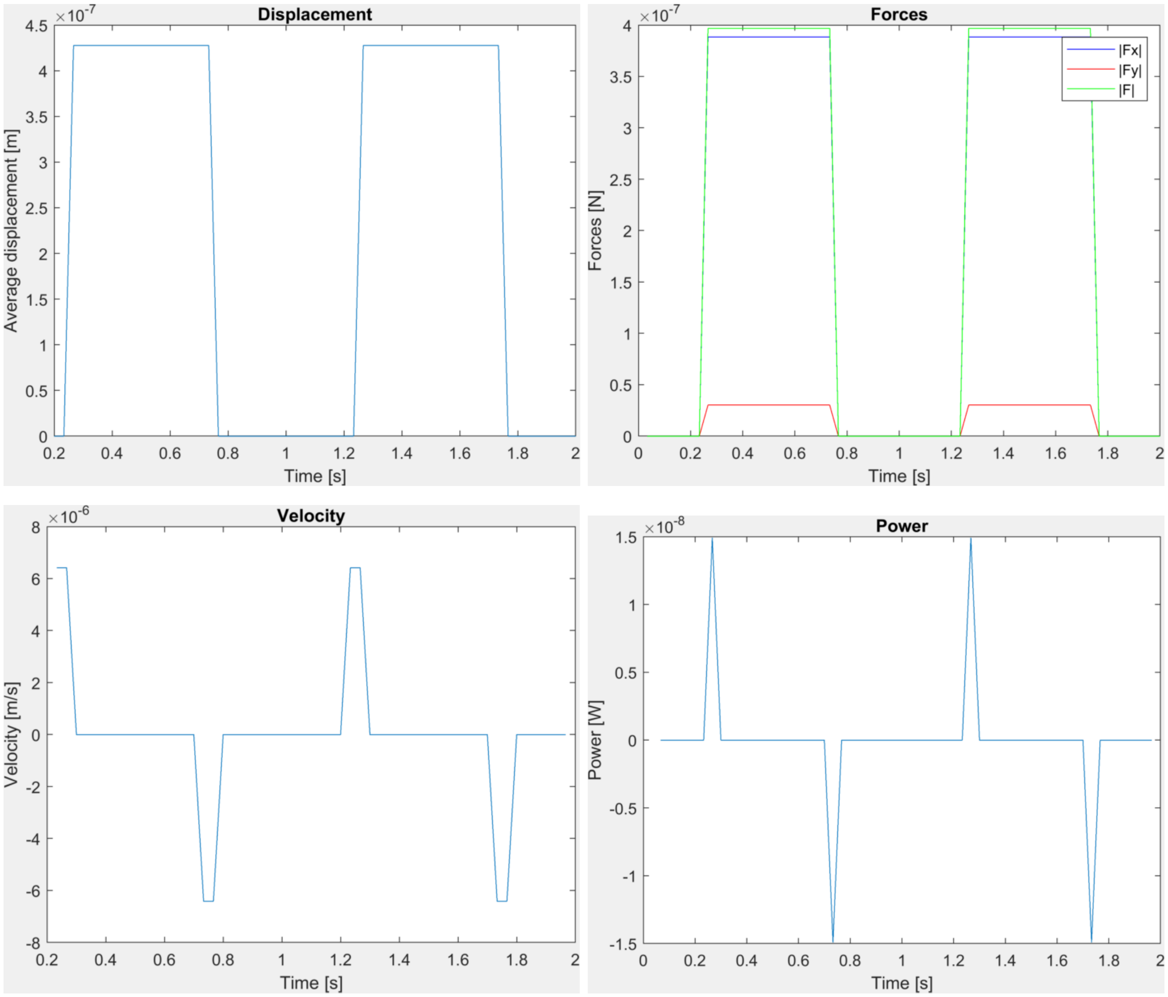

Figure S13: The temporal profile of the force dipole is user defined as a triangle (a), square (b) or following a user-define function (not shown). **a)** The displacement and force are accurately recovered in time. **b)** The square signal is accurately recovered without distortion in time. The velocity and power capture the abrupt change as single point peaks.

a

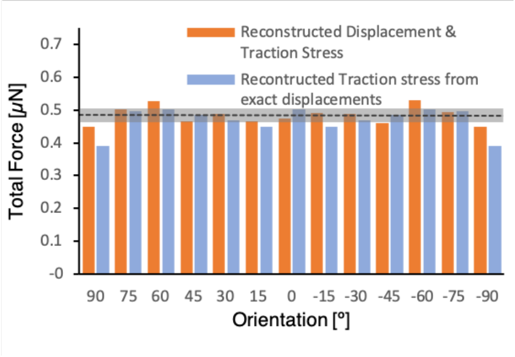

b

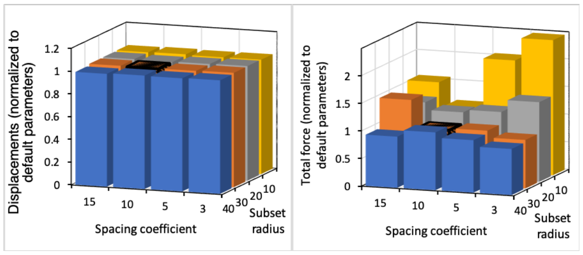

Figure S14 Cell orientation and analytical parameters impact on the accuracy of TFM analysis A) Total force recovery at slightly differs at various cell orientations when computed directly from the simulated displacement or from the virtual video using the TFM algorithm developed in our lab.[5]. **B)** PIV processing parameters impact on the accuracy of the calculation of average microsphere displacement and total force.

CONTRAX

STREAMLINED TFM GUI USER GUIDE

This document is a user guide for ContraX. It serves as reference and an introductory getting-started guide.

TABLE OF CONTENT

Requirements, Installation and Comments **1**

1. Requirements: 1
2. Installation: 2
3. Comments: 2

   Code details: 2

   Usage details: 2

Step-by-Step User Guide **3**

1. Initialization Panel: 3
2. Displacement PAnel: 11
3. Traction Force Panel 16
4. Results Panel 19

Output of computation **24**

REQUIREMENTS, INSTALLATION AND COMMENTS

1. REQUIREMENTS:

- ContraX should work without problem on computers equipped with a minimum of 8 GB RAM memory and 50GB disk space.
- ContraX has been tested for Windows 10, Windows Server 2018 and MacOS X 10.14.6.
- ContraX Streamlined TFM GUI code has been developed using Matlab R2018b.

2. INSTALLATION:

- Download Streamlined TFM GUI from our GitHub repository:
- Save source code on a disk that has enough free space to allow for analysis of videos. A disk with a fast read-write speed will perform best.

3. COMMENTS:

CODE DETAILS:

- Forward compatibility with Matlab and OS will depend on the changes made at that level. Updates will be provided on our GitHub to the best of our abilities.
- On recent versions of Matlab, the GUI produces a variety of warnings related to some of the packages. These can safely be ignored.
- The code uses some third-party packages, that are included in our repository release and do not require specific installation. Here is the complete list:
  - Ncorr1: DIC algorithms.
  - Bio-Formats for Matlab[2]: reads .czi image and video formats. This package is regularly updated to adapt to changes in proprietary formats. An update to a more recent version may be downloaded in the future.
  - xlwrite[3]: write results to Excel files.
  - peakdet[4]: determine peaks on noisy curves.
  - statusbar[5] and enableDisableFig[6]: freeze the windows during calculations and estimate runtime.
  - freezeColors[7] and COLORMAP and COLORBAR utilities[8]: display overlays on figures.
  - Exportfig[9]: flexible plot exports.[1]
- It is important to leave the functions and the file structure unaltered. Matlab must have writing rights to the working repository.

USAGE DETAILS:

- Computing time is reduced with the use of parallel processing; hence, multicore CPU machines will perform better.
- Parallel processing is used in part of the code that loop over the stack of video to analyze and well as in some function that perform analysis at the frame level. Hence, some of the parallel processing benefits only become evident during the processing of multiple videos and/or long videos.

---

1 http://ncorr.com
2 https://www.openmicroscopy.org/bio-formats/downloads/
3 http://www.mathworks.com/matlabcentral/fileexchange/38591-xlwrite--generate-xls-x--files-without-excel-on-mac-linux-win
4 http://www.billauer.co.il/peakdet.html
5 http://www.mathworks.com/matlabcentral/fileexchange/14773-statusbar
6 http://www.mathworks.com/matlabcentral/fileexchange/15895-enable-disable-figure
7 http://www.mathworks.com/matlabcentral/fileexchange/7943-freezecolors---unfreezecolors
8 http://www.mathworks.com/matlabcentral/fileexchange/24371-colormap-and-colorbar-utilities--jul-2014-
9 http://www.mathworks.com/matlabcentral/fileexchange/727-exportfig

- When large batches of videos are analyzed, memory and disk space requirements will increase accordingly. During execution, a significant amount of temporary data as well as results files and variables are written to the disk or kept in RAM memory. Thus, it is advisable to have at least a few tenths GB of free disk space. In the PIV step, a tick mark option is available to relieve memory pressure for the processing of large batches of videos. In this case, some of the PIV output heatmap figures are not saved, but no data loss occurs. If out-of-memory error occurs, try to rerun the analysis with fewer videos at first.
- Calculations can be stopped at any time using the Matlab keyboard shortcut: ctrl + C key combination.

STEP-BY-STEP USER GUIDE

ContraX Streamlined TFM BUI is launched by executing tfm_gui_main.m.

The analysis is split into four sequential parts, each of them requiring user setup and review. Steps which are not accessible at a current stage are greyed out, steps which have been completed show up green.

We strongly recommend to close Matlab between subsequent executions of video batches, as some of the temporary data are saved as app data and may not be cleared without restarting Matlab.

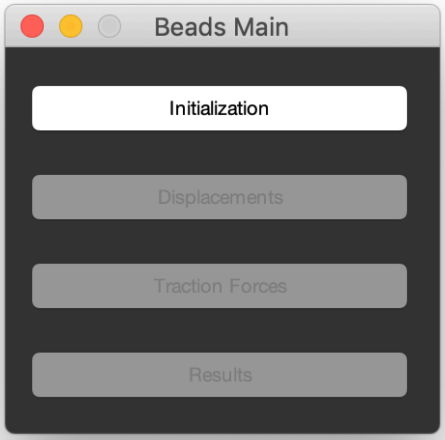

FIGURE 1 STREAMLINED TFM GUI MAIN CONTROL WINDOW.

1. INITIALIZATION PANEL:

The initialization panel is where the videos to analyze area loaded and image preprocessing is performed. Some image parameters also need to be provided.

1. In the main panel (Figure 1), launch the initialization panel by clicking on the *Initialization* button (Figure 2).

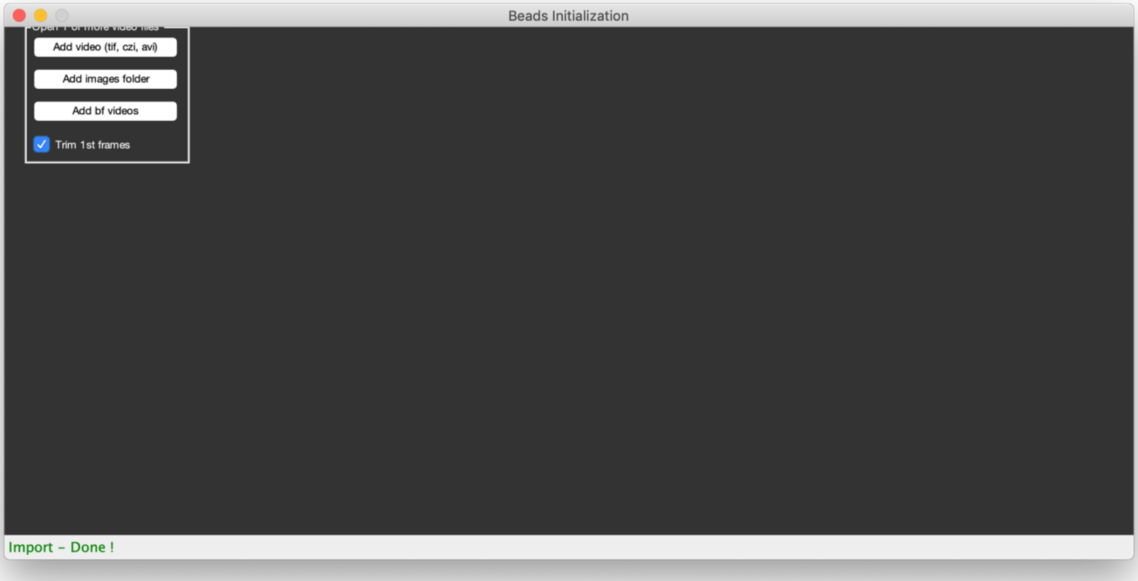

FIGURE 2 INITIALIZATION PANEL

2. Load videos of the fluorescent beads using Add video (tif, czi, avi) button. Use to file explorer to locate the video of interest, toggling for the desired file extension type. Several videos can be selected and loaded at once, but they must ideally reside in the same folder.
    - Sometimes, we experienced that the first frame of the .czi video was not acquired correctly by our microscope camera, therefore we implemented an option to *trim the first frame* of the video. This tick mark must be selected prior to import the videos.

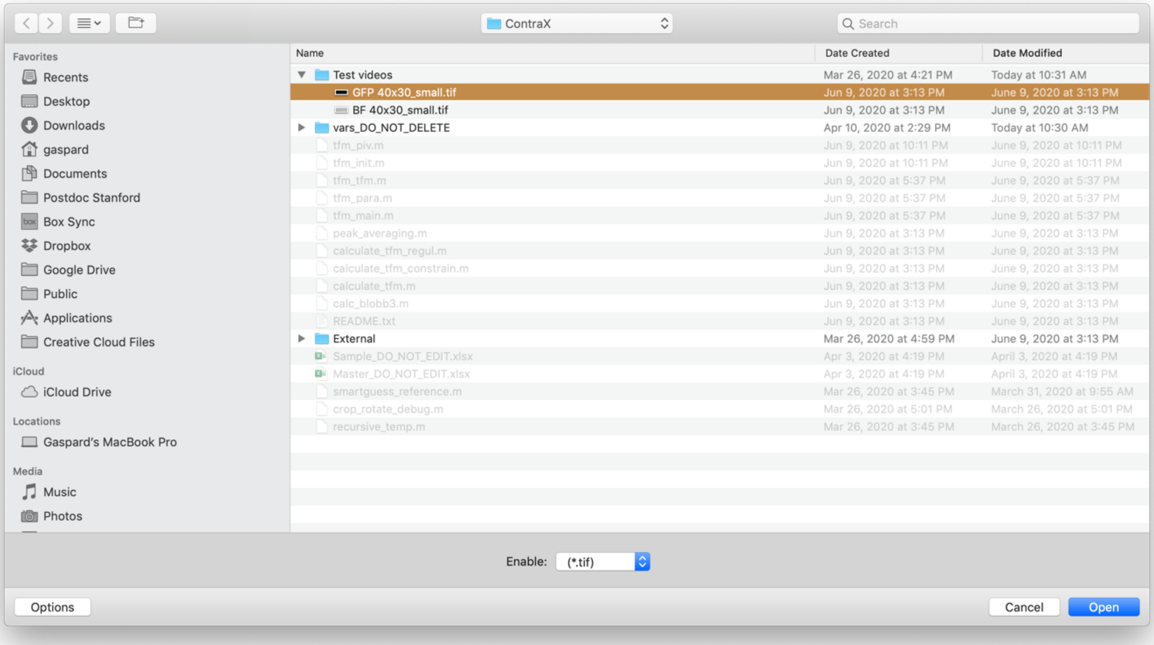

FIGURE 3 SELECT VIDEOS TO ANALYZE

- If cell outline masks were defined for some of the videos being loaded during a prior analysis, the following pop-up will appear asking if the user wants to reimport these masks automatically. This save precious time if a batch of cells needs to be analyzed a second time.

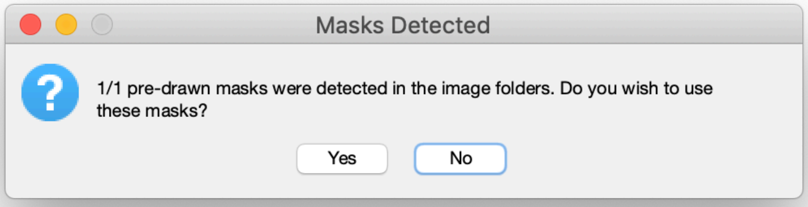

FIGURE 4 CHOOSE TO IMPORT MASK THAT WERE DEFINED DURING A PRIOR ANALYSIS

- Multichannel .czi video can be loaded and a pop-up window will ask to assign each channel to the corresponding TFM or brightfield/fluorescence image for cell outlining (Figure 5).

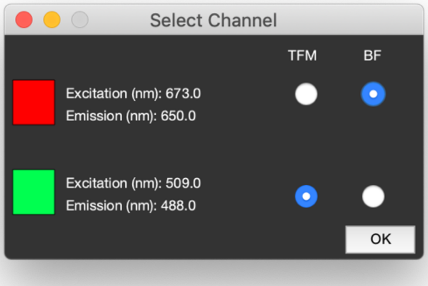

FIGURE 5 MULTICHANNEL .CZI FILES CAN BE IMPORTED AND EACH CHANNEL MUST BUST ASSIGNED TO TTFM OR BRIGHTFIELD/FLUORESCENCE FOR CELL OUTLINING

3. After loading, the list of loaded videos and a preview frame of the fluorescent beads video are displayed. If masks are loaded from a prior analysis, the cell outline and encompassing analysis ellipse are displayed in overlay in red and blue, respectively. If no mask was loaded, the preview frame does not display any outline.

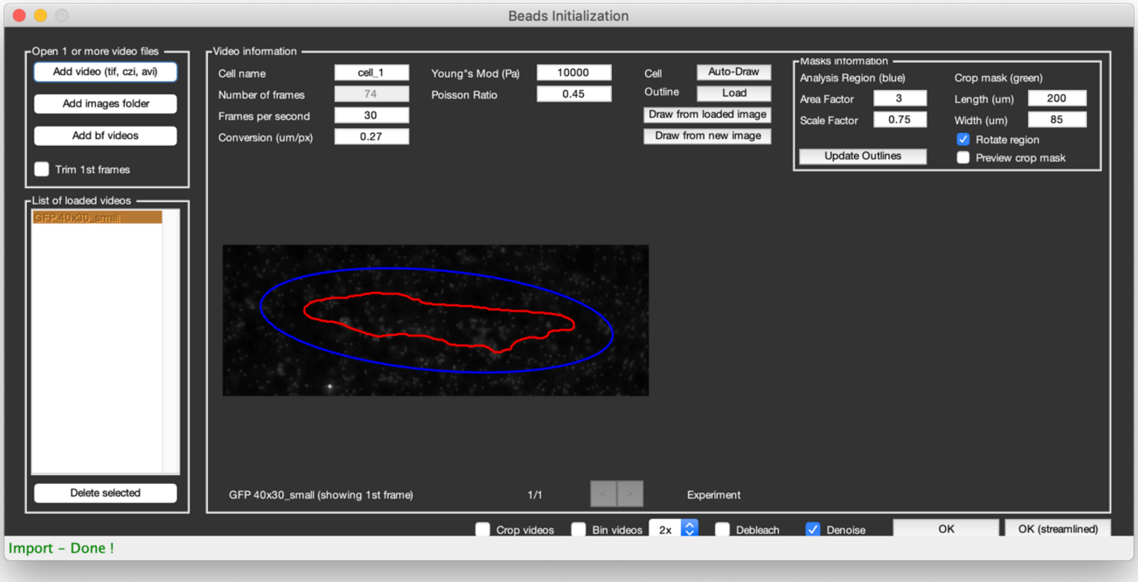

FIGURE 6 ONE VIDEO WAS LOADED WITH A PREVIOUSLY DEFINED MASK AND A PREVIEW IS SHOWN

4. Load brightfield/fluorescent image or video to define the cell outline by clicking on the *Add bf video* button.

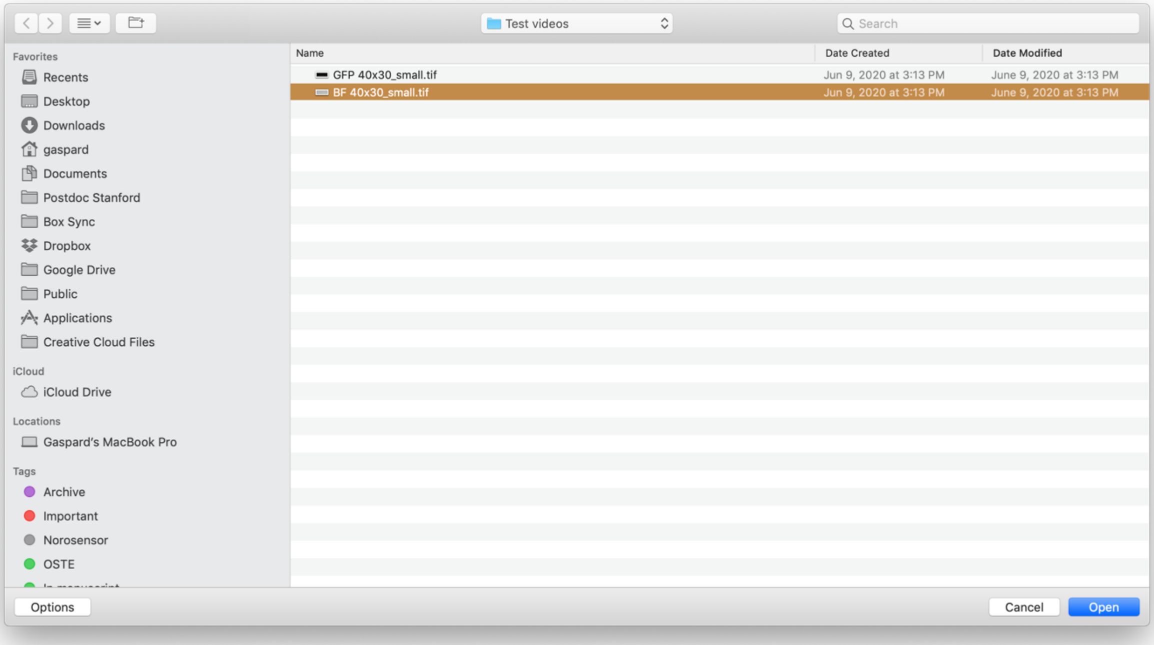

FIGURE 7 SELECT BRIGHT FIELD IMAGE FOR CELL OUTLINING

5. Draw a cell outline for each of the loaded videos: 4 options are available and must be performed for each video:
    a. *Auto-draw*: The software uses a built-in algorithm to automatically detect cell objects and outline in the brightfield/fluorescent image for each video sequentially. The user just needs to select in each black and white mask image which object to outline (Figure 8). Once the stack of videos is processed, the user can refine the outlines manually using the option below. The process can also be programmatically fully automated (from within the code), but, as currently implemented in the code, will only be able to select the one object detected in the center of the image frame.

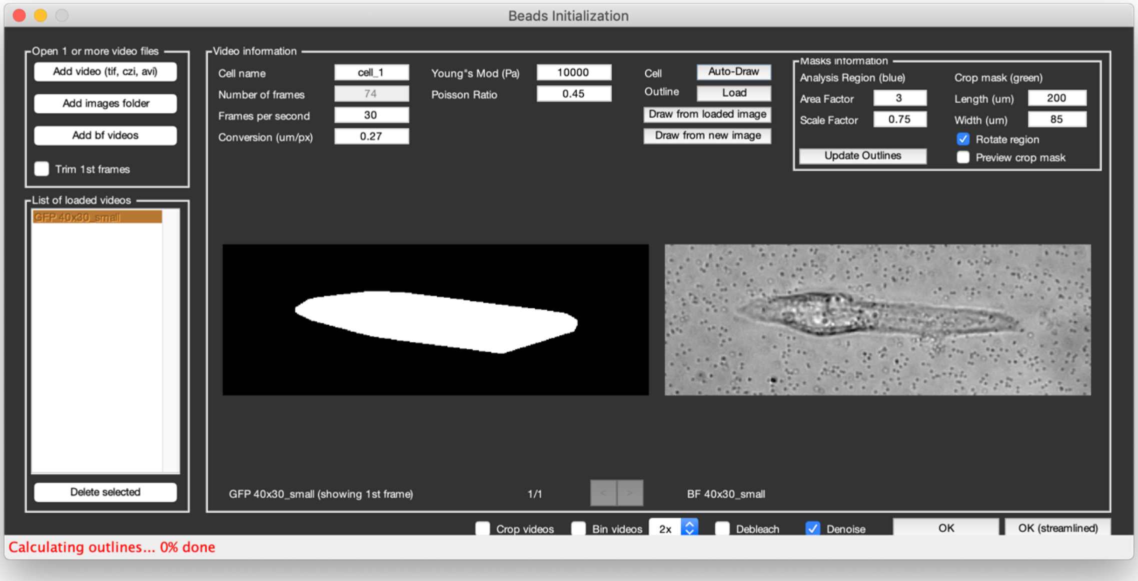

FIGURE 8 AUTO-DRAW CELL OUTLINE AUTOMATICALLY DETECTS THE CELL OUTLINE AND USER JUST NEED TO SELECT WHICH OBJECT TO ANALYZE BY DOUBLE CLICKING IN THE OBJECT

b. *Load*: Load a predefined outline saved a binary mask *.mat* file.

c. *Draw from loaded image*: Let the user draw a mask by clicking around the cell outline on the previously loaded brightfield / fluorescence image (Figure 10).

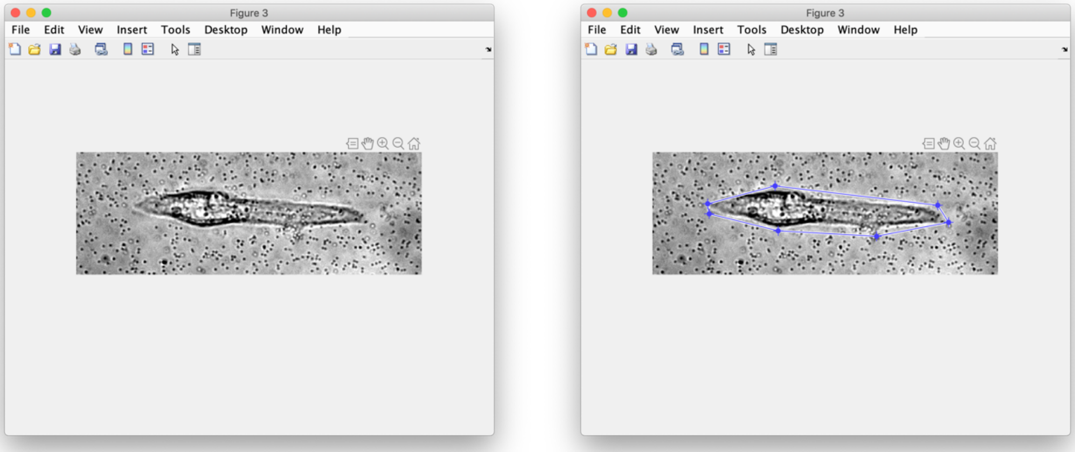

FIGURE 9 DRAW THE CELL OUTLINE BY CLICKING AROUND THE CELL

   d. *Draw from new image*: Let the user load another brightfield / fluorescence image to draw a cell outline on, in a similar way as c). This will replace the brightfield / fluorescence image previously loaded for a corresponding fluorescent beads video.

6. The cell outline is displayed in red and the analysis area in blue (Figure 10). The size of the analysis area can be changed, see step 9.

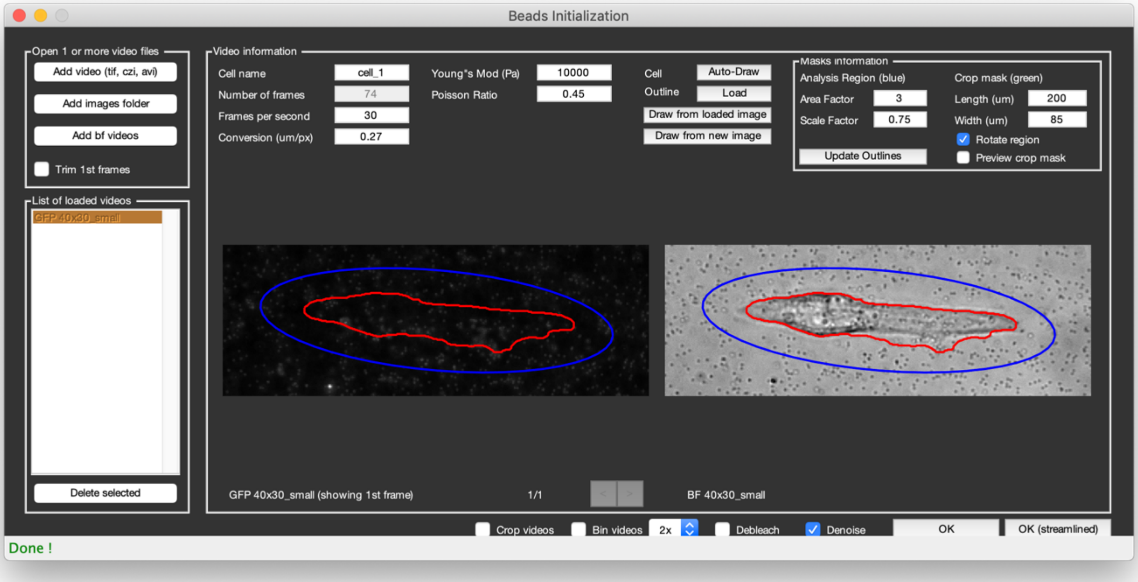

FIGURE 10 PREVIEW OF THE CELL OUTLINE AFTER AUTO-DRAW / DRAW / LOAD IS PERFORMED

7. Make sure that all cells have been outlined, that the video parameters, *i.e., Frame per second, Conversion (um/px), Young's Modulus (Pa),* and *Poisson ratio* are correct for each video. Note: the default values can be edited in the code prior to start.
8. Make sure to use the appropriate *Analysis region (blue)* parameters for each video. The *Area Factor* controls the area of the blue ellipse to be x time larger than the ellipse that best fit the cell outline. The *Scale Factor* enables to control the aspect ratio of the ellipse, *i.e.,* a scale factor < 1 makes the ellipse less elongated, which allows to avoid having the ellipse going much out of frame in the case an edge of the video frame is too close to the cell. In some cases, that could result in analysis bias or unwanted noise from edge effect in the PIV step.
9. Select the size of the cropping mask if cropping of the video is desired. Note that enough space between the cell and the video frame edges is necessary to capture gel deformation that can propagate far from the cells in the case of a soft hydrogel formulation. Select if the cropping region should also rotate the image while cropping. Click on the *Update Outline* button to refresh the outlines preview (Figure 11).

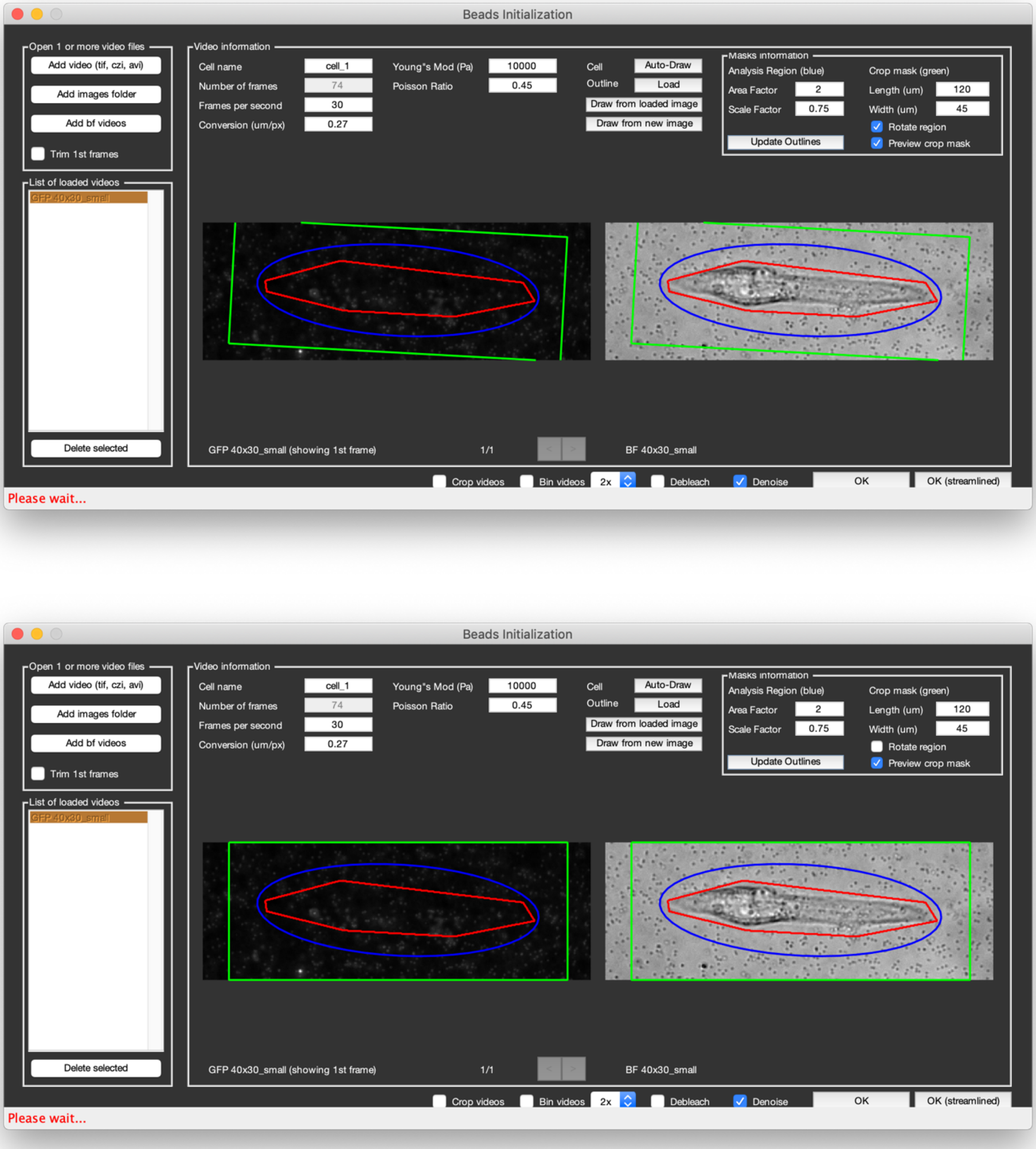

FIGURE 11 CROPPING OUTLINE IS SHOWN IN GREEN, WITH OR WITHOUT ROTATION, AS SELECTED BY THE USER

10. Before to finalize the initialization, verify the parameters a last time and decide whether to *Crop*, *Bin*, *Debleach* or *Denoise* the videos (Figure 12).
11. Finally, decide to proceed with the analysis step-by-step, which allows for editing the defaults analysis parameter in subsequent steps, by clicking on the *OK* button, or in streamlined mode, which will proceed with the analysis using the default parameters (editable in the code), by clicking on *OK (streamlined)*.

- Warning is shown before to proceed with Streamlined analysis. Close it to proceed (Figure 13).

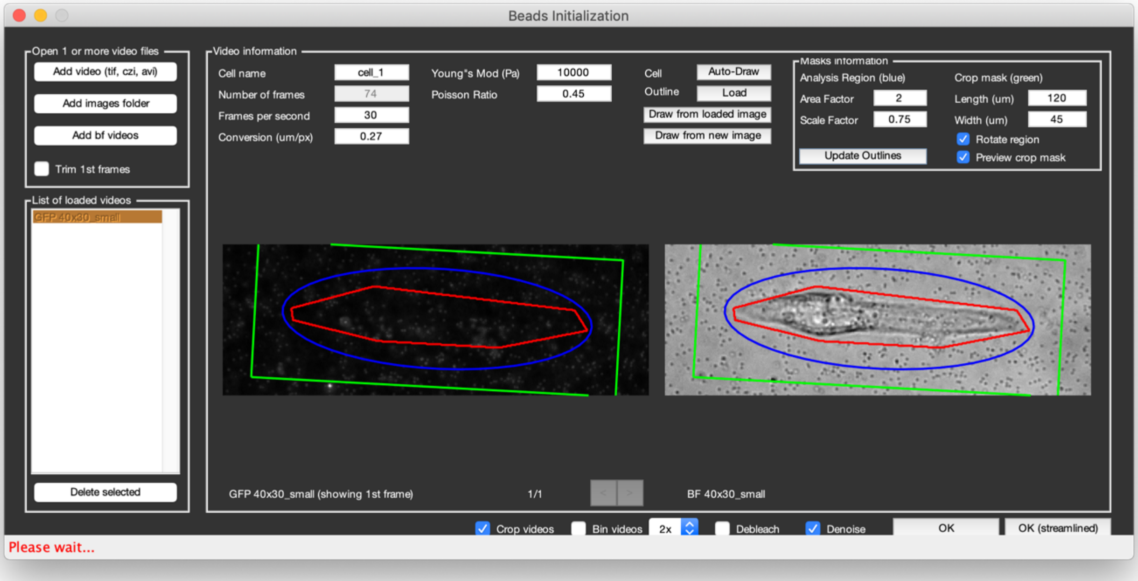

FIGURE 12 BEFORE TO FINISH, SELECT WHETHER TO PERFORM CROPPING, BINNING, DEBLEACH AND DENOISING

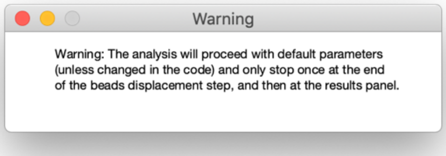

FIGURE 13 A WARNING IS SHOWN BEFORE TO PROCEED WITH STREAMLINED ANALYSIS. CLOSE IT TO PROCEED.

2. DISPLACEMENT PANEL:

The displacement panel is where the PIV analysis of the gel deformation is performed by calculating the displacement of the fluorescent beads using the Ncorr algorithm. This step uses parallel processing over the videos stack and video frames.

1. In the main panel (Figure 14), launch the initialization panel by clicking on the *Initialization* button (Figure 15).

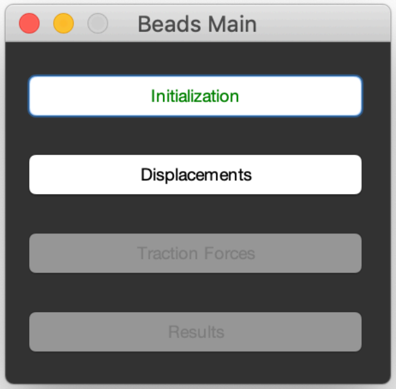

FIGURE 14 AFTER INITIALIZATION IS COMPLETED, THE DISPLACEMENT STEP IS ENABLED

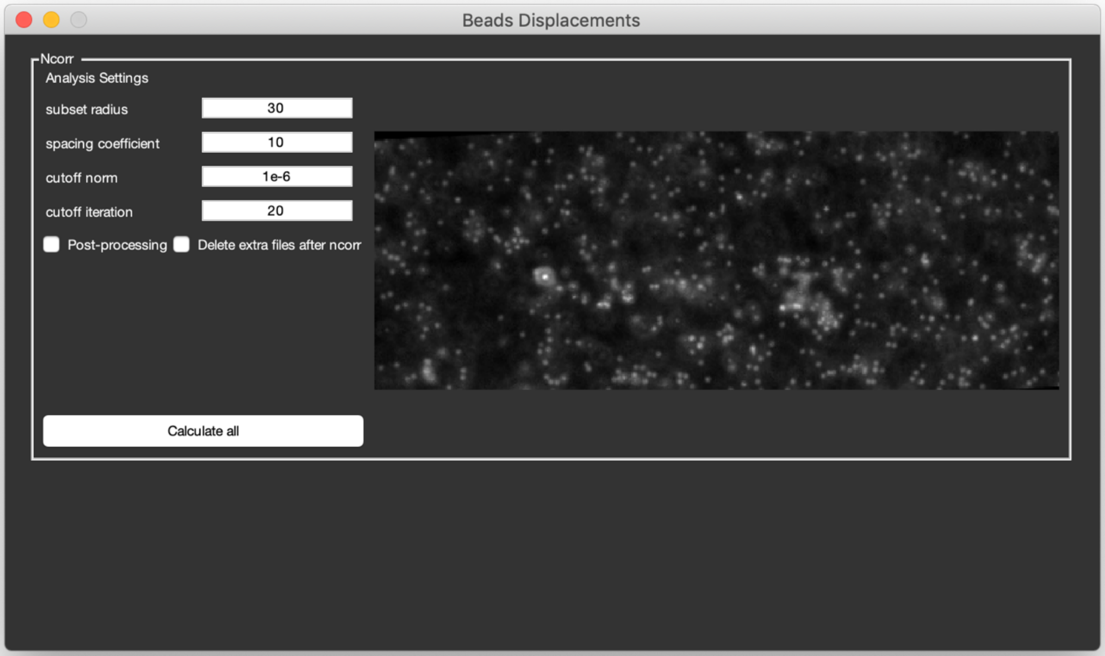

FIGURE 15 DISPLACEMENT PANEL

2. The first step calculates the beads displacement with the Ncorr PIV algorithm and requires choosing values for the processing parameters:
   - *Subset radius*: controls the size (in pixels) of the image windows whose position is tracked using the beads. The adequate value depends on the experimental conditions, such as the beads density, depth of field of the objective used for imaging, image noise, etc.
   - *Spacing coefficient*: controls the spacing (in pixels) between each image windows analyzed. A small value will result in a measurement of the displacement at many spatial locations while a large value will spatially down sample data and underestimate the hydrogel deformation.
     i. Note: Both values will strongly influence the computing time, and it is important to always use the same values for all the data analysis to obtain comparable results.
   - *Cutoff norm* and *cutoff iteration* control the accuracy of analysis.
3. Select whether post-processing is desired, in which case additional parameters must be chosen to filter data during a post-processing step. Such post-processing must be used with caution and with rigorously the same parameter values for experimental consistency (Figure 16).
4. Select whether to delete extra files after the Ncorr PIV calculation step to preserve memory. In this case, it is not possible to save the .png images and .mat displacement field data of the PIV analysis results. It can be a necessary choice depending on the hardware used and the number of videos processed in the batch. If selected, it is not possible to rerun this step without going through the *Initialization* panel again to reload some of the data.
5. Once parameters are chosen, launch the PIV calculation by clicking on the *Calculate all* button.

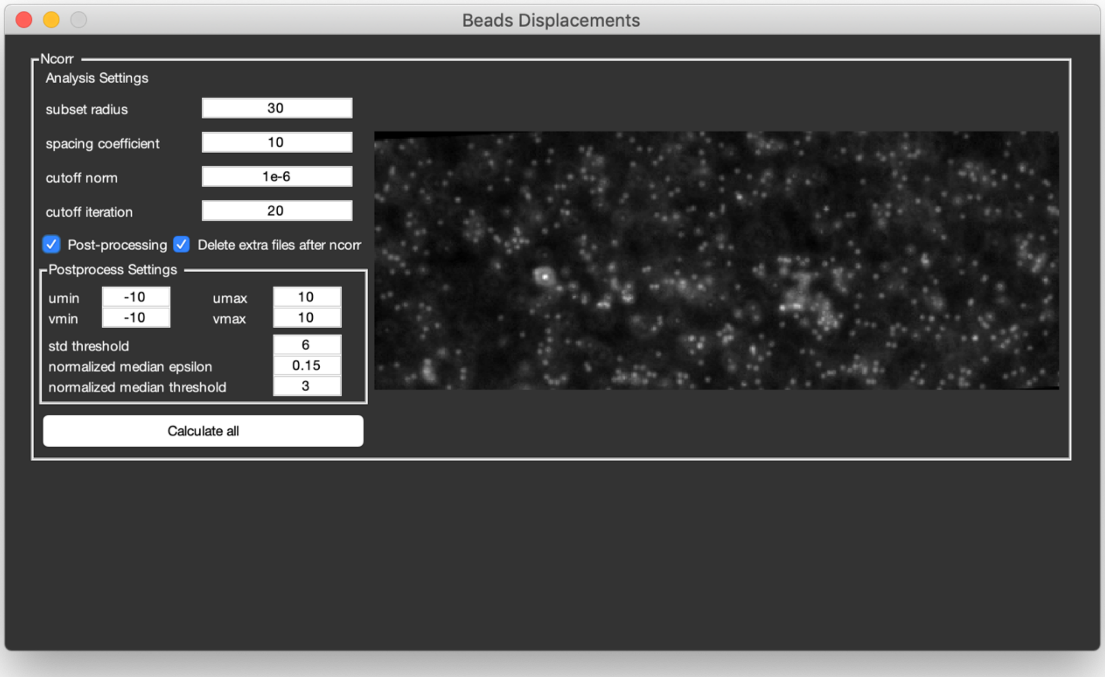

FIGURE 16 POST-PROCESSING CAN BE PERFORMED IN POST-PROCESSING. MUST ONLY BE USE AFTER TESTING AND WITH CONSISTENCY.

6. After the PIV step is performed, the displacement is automatically referenced to the most relaxed image frame. The average displacement trace is displayed for each video for review. (Figure 18) It must be verified for each video and eventually manually corrected. It can happen that the algorithm does not adequately identify the relaxed frame. In some instance, a contracting frame is mistaken for the relaxed frame, and the curve in up-side-down, the arrow directions in the image preview helps verify that the displacement points inwards towards the center of the cell. In some instance, *i.e.,* weak contraction and/or noisy data, the frame identified by the algorithm is not ideal and a manual identification can sometimes improve the data. If unsure, proceed with the analysis and decide whether to exclude or reprocess the data at the results step. To adjust the frame referencing, either enter the frame numbers for a (the most) relaxed and contracted frames or click on the respective *Pick* button. The latter option opens a pop-up with the average displacement curve where the reference point can be picked by clicking on the curve (Figure 17).

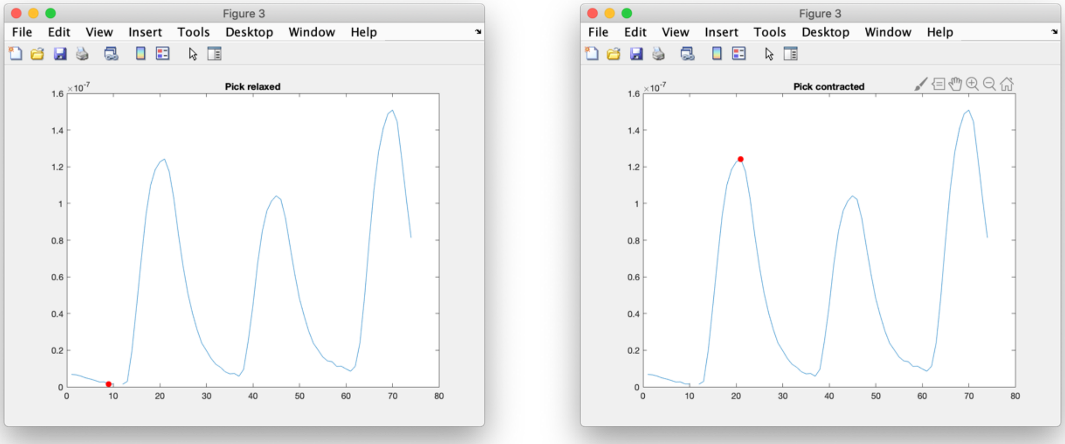

FIGURE 17 TO ADJUST THE FRAME REFERENCING MANUALLY, SELECT THE RELAXED AND CONTRACTED FRAMES IN THE MEAN DISPLACMENT CURVE

7. Once reviewed, select *Calc. Displacement* to calculate the referenced displacement.

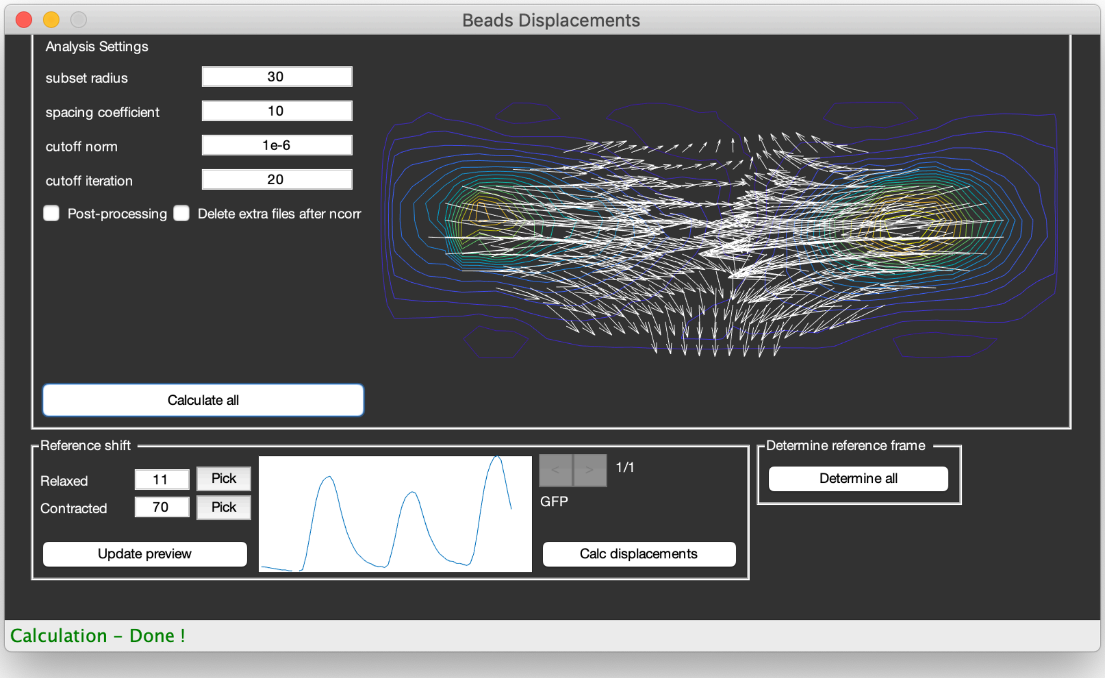

FIGURE 18 AFTER THE PIV CALCULATION STEP, THE FRAME REFERENCING MUST BE REVIEWED AND MANUALLY CORRECTED IF NECESSARY.

8. Once reviewed, select *Calc. Displacement* to calculate the referenced displacement (Figure 18).
   - Note: If streamlined analysis is selected in the initialization panel, the computation proceeds automatically up to this point. The referenced displacement is calculated but we recommend verifying the referencing (step 6) and recalculating the referencing if any of the reference frame is changed (step 7). If no change is made, proceed with saving the data directly.
9. Select what data to save and click on *OK* to proceed (Figure 19).

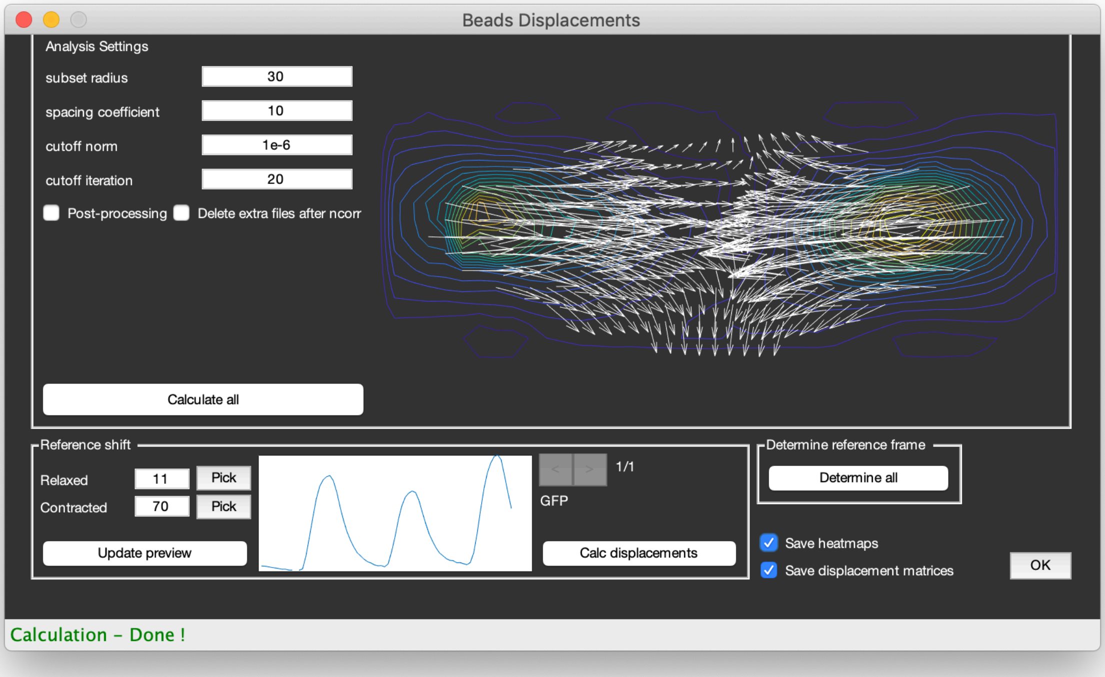

FIGURE 19 ONCE THE REFERENCED DISPLACEMENT ARE CALCULATED, SAVE THE DATA AND CONTINUE WITH OK.

3. TRACTION FORCE PANEL

1. In the main panel (Figure 20), launch the initialization panel by clicking on the *Initialization* button (Figure 21).

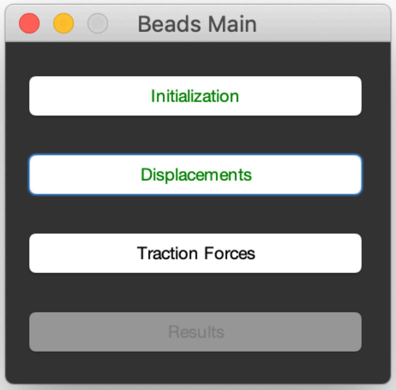

FIGURE 20 AFTER THE DISPLACEMENT STEP IS COMPLETED, THE TRACTION FORCE STEP IS ENABLED

2. When the *Traction Force* panel is launched, the calculation of the estimation of the regularization parameter is performed automatically.
3. Update the material parameters for each video if necessary (that is normally already set and verified in the *Initialization* panel).
4. Choose whether to proceed with *unconstrained analysis* or *constrained analysis* of the traction stress. *Unconstrained analysis* computes the traction stress for the whole frame, while *Constrained analysis* will assume zero traction stress outside of the analysis area (blue ellipse in the *Initialization* panel). The choice is left to the user to be made and more information can be found in Sabass et al. (2008), but this choice should be maintained the same for all videos analyzed and dataset to be compared.
5. Proceed with the computation of the traction stress by clicking on the *Calculate all* button.

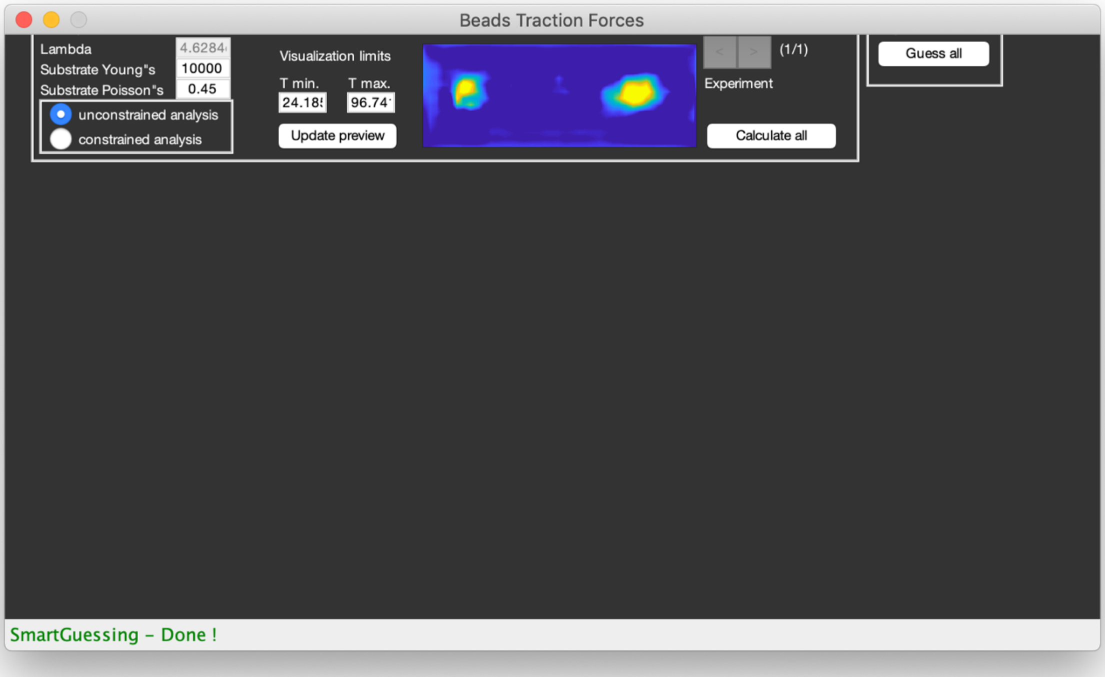

FIGURE 21 WHEN THE TRACTION FORCE PANEL IS LAUNCHED, THE ESTIMATION OF THE REGULARIZATION PARAMETER (LAMBDA) IS PERFORMED AUTOMATICALLY

6. Select what data to save and click on *OK* to proceed (Figure 22).

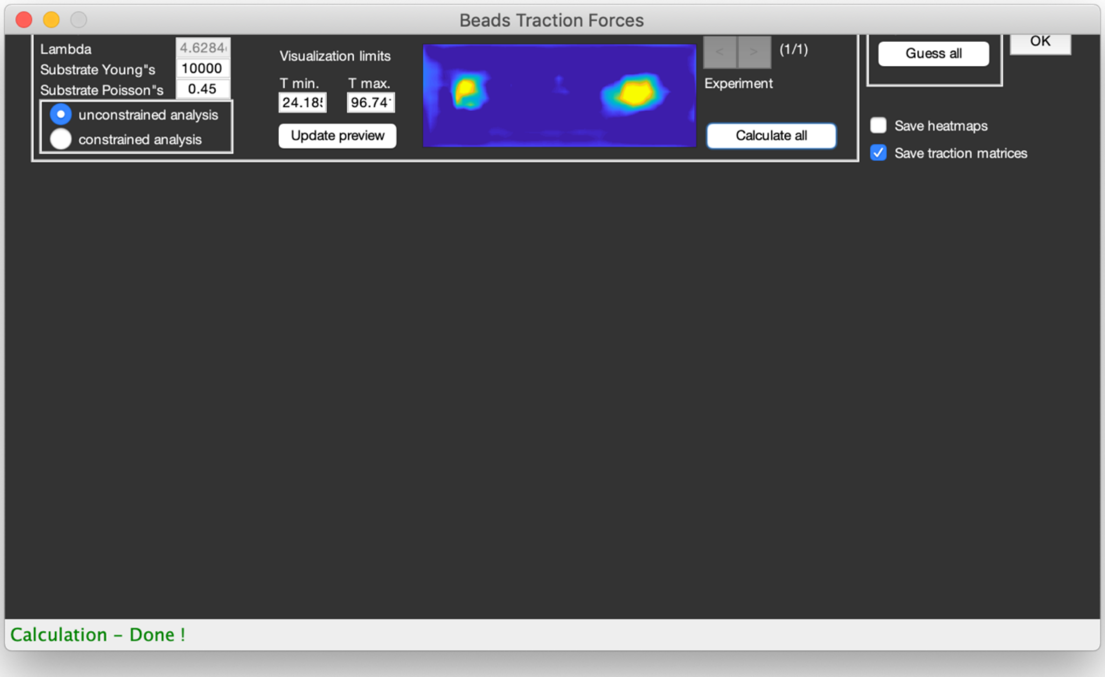

FIGURE 22 SAVE DATA AND PROCEED WITH OK.

4. RESULTS PANEL

1. In the main panel (Figure 23), launch the initialization panel by clicking on the *Initialization* button (Figure 24).

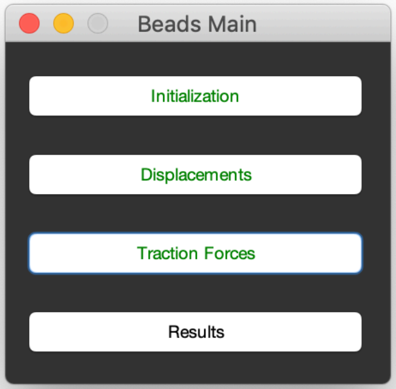

FIGURE 23 AFTER THE TRACTION FORCE STEP IS COMPLETED, THE RESULTS PANEL IS ENABLED.

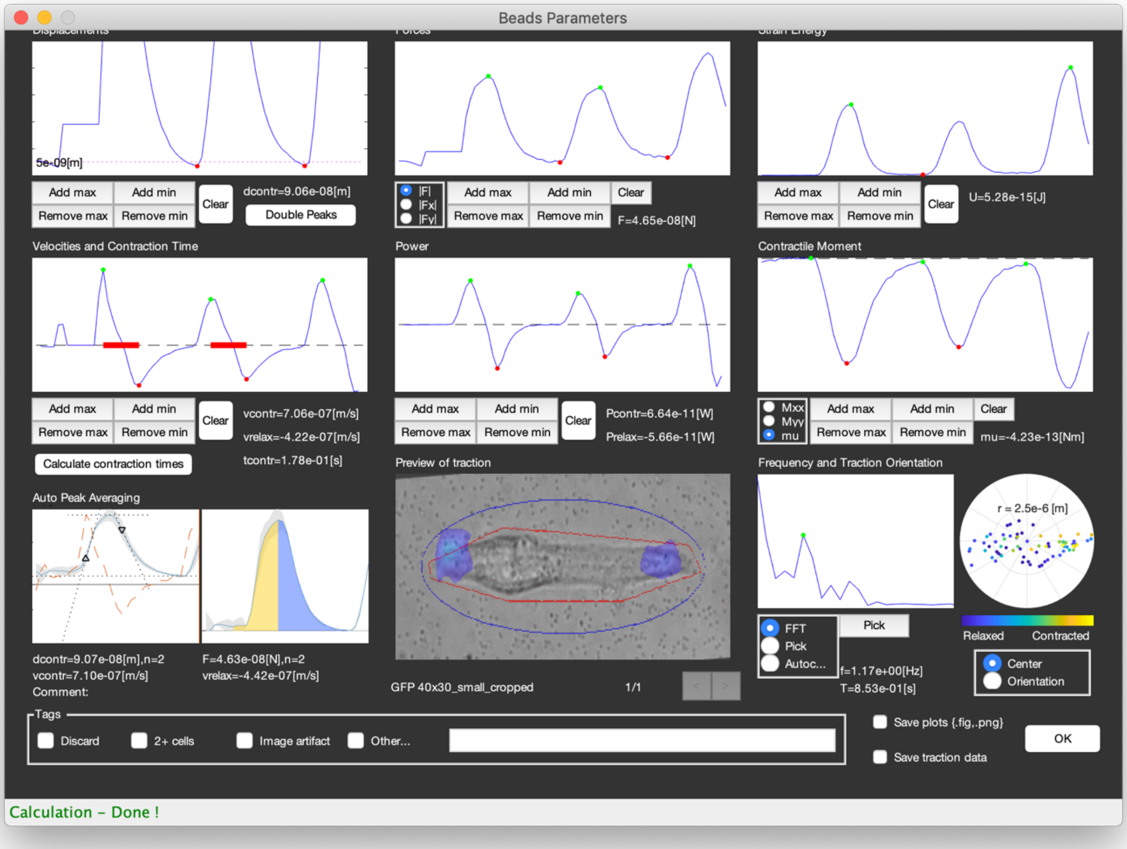

FIGURE 24 THE RESULTS PANEL DISPLAY MOST OF THE MEASURED PARAMETERS AND A PROVIDE A VISUAL SUMMARY OF THE ANALYSIS FOR EACH CELL.

2. When the results panel is launched, the software calculates all the contractile parameters for each cell. The results panel displays a summary of the analysis for each cell, with, top 6 panels left-to-right row-by-row, the time traces of the:
   - *Average bead displacement* (*i.e.*, the average value of the displacement amplitude within the analysis area (blue ellipse)),
   - *Total force* (*i.e.*, the surface integration of the traction stress amplitude (or along and perpendicularly to the cell main axis) within the analysis area (blue ellipse),
   - *Strain energy* (*i.e.*, measured within the analysis area (blue ellipse),
   - *Velocities* and *Contraction time* (*i.e.*, the maximum and minimum velocities of contraction calculated as the derivative of the average bead displacement, and the duration of contraction measured as the duration between maximum and minimum contraction velocities),
   - *Contraction Power* (*i.e.*, the product of the contraction force and velocity),
   - *Contractile moment* (*i.e.*, the measure of the strength of the force dipole create by the cell.

  - For each of these parameters, the peaks of interested are automatically selected and can be manually added or remove by the user.
  - In addition, if users are interested to capture addition data regarding the presence of double peaks, due to contraction twitch or cell doublet, this can be done by selecting the *Double peak* button and adding the relevant parameter in the pop-up panel.
3. The bottom row figure shows left to right:
  - The results of the automated peak detection and averaging for the *Average displacement* and Contraction velocities, and of *Total force* with the area integrated for the computation of the *Contraction impulse*. Here, the peak detection is entirely automated, and the other parameters are also calculated in a similar way in the background. The user has no direct access to the data which remove the risk for user bias in the peak selection. The number of peaks that are detected and used for the averaging is mentioned below the figure with the valued of the measured amplitude.
  - A .gif video showing the contraction stress superimposed onto the brightfield/fluorescent preview image for each cell. This provide the user with rapid way to further assess the qualitative value of a measurement and potentially flag a cell using the predefined flags tick boxes and comment field.
  - A radio-button-switchable figure showing:
    - *FFT*: the FFT transformation of the A*verage displacement* used to measure the C*ontraction frequency*, enabling the user to manually refine the peak selection.
    - Pick: the user can measure the contraction frequency by picking peaks manually in the *Average displacement* curve, as well.
    - Autocorrelation: the autocorrelation of the *Average displacement* trace used to automatically identify and average the contraction peaks, which provide an additional way to qualitatively assess the quality of the automated analysis.
  - A radar plot showing the:
    - *Position* of the center of pression with respect to the center of the cell and provides a measure of the contraction coordination and homogeneity within the cell.
    - *Orientation* of the contraction dipole with respect to the cell morphology main axis, which provides a measure of the alignment of the contraction dipole with the cell geometry. In patterned elongated cell, this angle should remain minimal, while in non-patterned cells, the angle may vary in time.
4. *Tags*: The user can select between four predefined tags to mark cell videos that may not match the user required data quality and a comment field. The flag value for each tag and the comments left by the users are saved in the results file and enable easy data curation post-analysis (Figure 25).
5. Once the results are verified for each cell, the user select what additional data to save and proceed with *OK* to finalize the analysis. Finally, the main panel can be closed, and the analysis is finished (Figure 26).

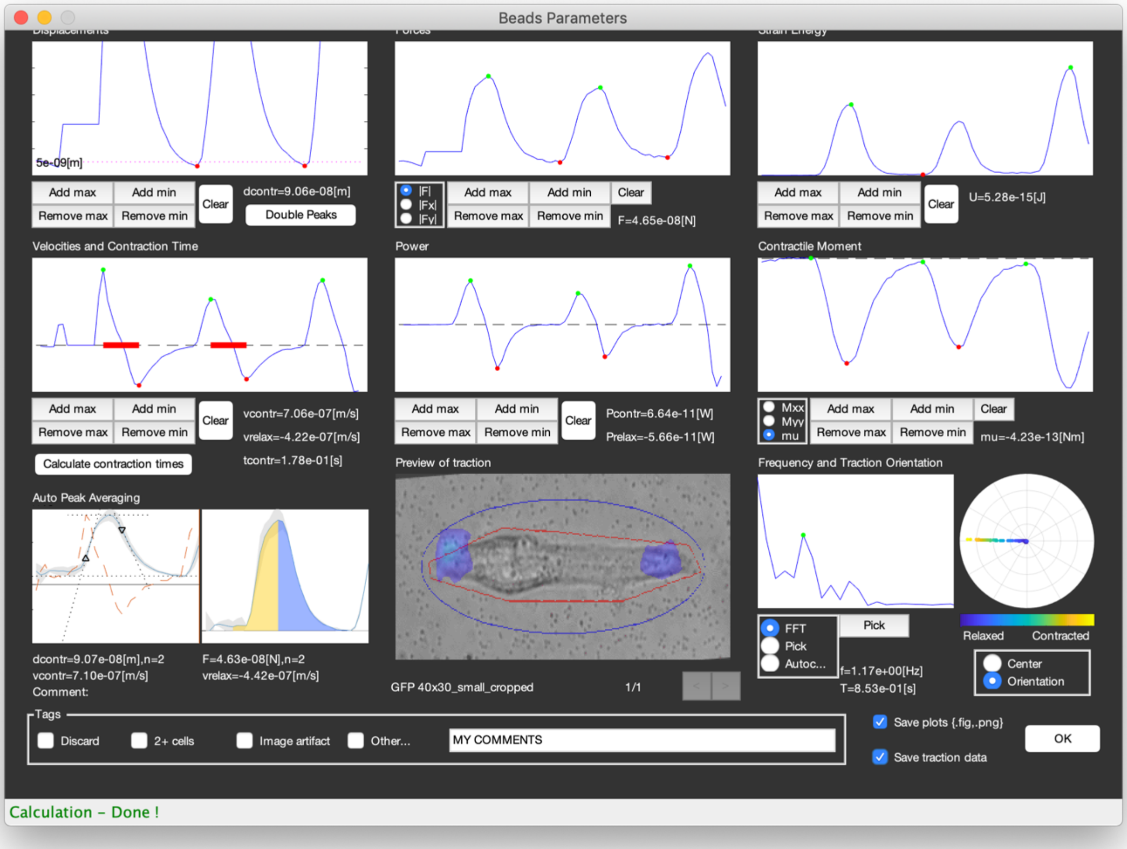

FIGURE 25 ONCE THE RESULTS ARE VERIFIED FOR EACH CELL, THE USER SELECT WHAT ADDITIONAL DATA TO SAVE AND PROCEED WITH OK TO FINALIZE THE ANALYSIS

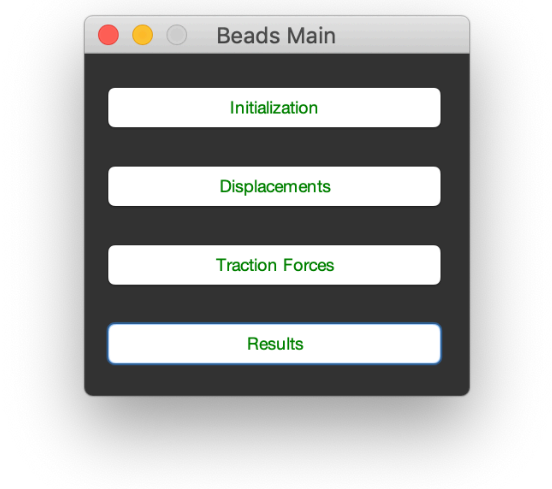

FIGURE 26 ONCE ALL THE STEP ARE COMPLETED, THE MAIN PANEL CAN BE CLOSE AND THE ANALYSIS IS FINISHED

OUTPUT OF COMPUTATION

The analysis produces a large amount of data and many output files and enough disk space is required.

The output file structure is as follow (Figure 27):

- If the videos are cropped and/or binned, this new version of the video is saved, and the original version is preserved on disk.
- A *Batch_results.xlsx* file is generated at the end of the results step, which combine the measured parameters for each of the videos analyzed during a batch analysis. The data for the manually selected peaks and for the automated peak averaging analysis are saved on separate tabs. This allows for rapid analysis and interpretation of data.
- A folder is created for each video loaded. A separate folder is created for the brightfield/fluorescent video/image if it has a different name than that of the corresponding beads video. For multichannel .czi, only one folder is created.
  - In the folder for the brightfield/fluorescent video/image, the cell outline mask only is saved (Figure 28).
  - In the folder for the beads video, the substructure shown in Figure 29 is generated:
    - *Plots*:
      - *Curve* plots: contains .png and .fig files of the results panel figures for easy and rapid review for each cell.

    - *Traction heatmaps*: contains .png images of the traction heatmap for each frame and enables to generate a movie of the traction stress.
- *Dataset*:
    - *Traction Data*: contains the full resolution interpolated x and y component of traction stress as .mat files.
    - *Traction Forces*: contains the raw traction stress data at the point of analysis, *i.e.,* at the position of each PIV window tracked at the PIV step.
    - *Displacements*: contains the raw data of the displacement at the point of analysis, *i.e.,* at the position of each PIV window tracked at the PIV step.
- A .gif file of the overlay of the traction stress over the cell image (same as shown in the *Results panel*)
- *Mask*: contains the mask of the cell outline as .mat file.
- *Results*: contains a .xlsx file with the complete results for each cell, including all the parameters save in the *Batch_results.xlsx* file and the time trace for all the parameters and the averages peaks curve.

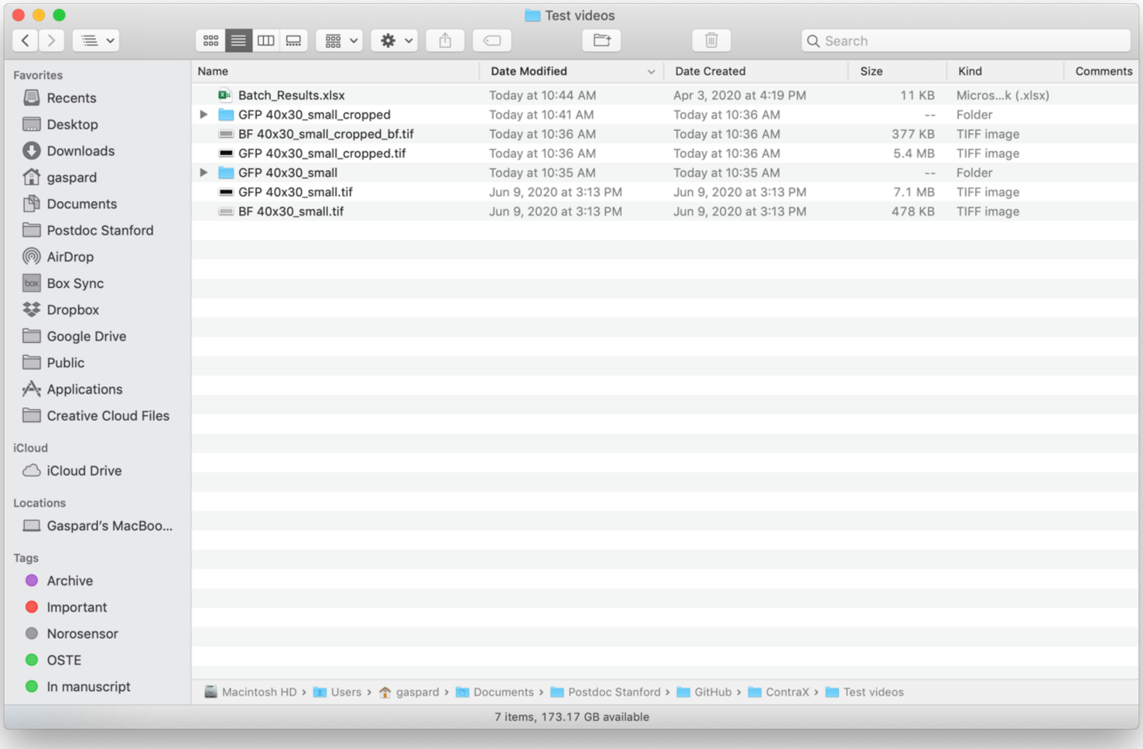

FIGURE 27 FILE STRUCTURE CREATED FOR EACH OF THE ANALYSED VIDEOS IN THE ROOT FOLDER

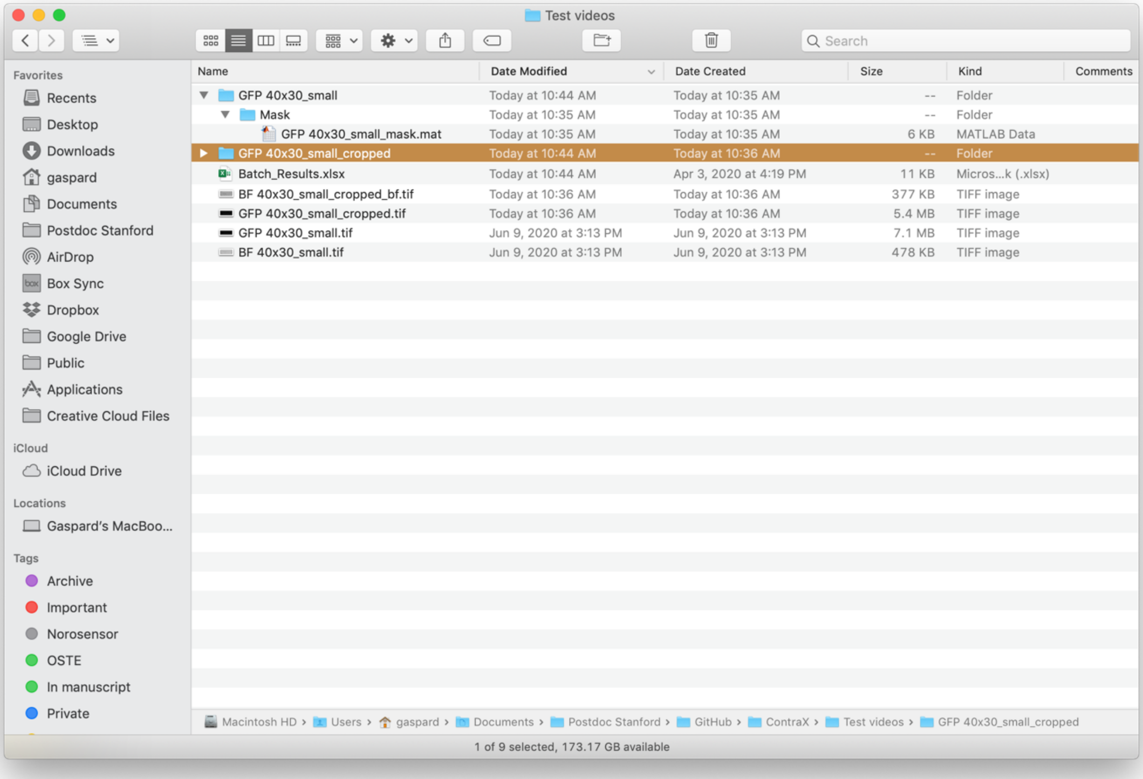

FIGURE 28 ONLY THE CELL OUTLINE MASK IS SAVED IN THE FOLDER NAME AFTER THE BRIGHTFIELD/FLUORESCENT VIDEO

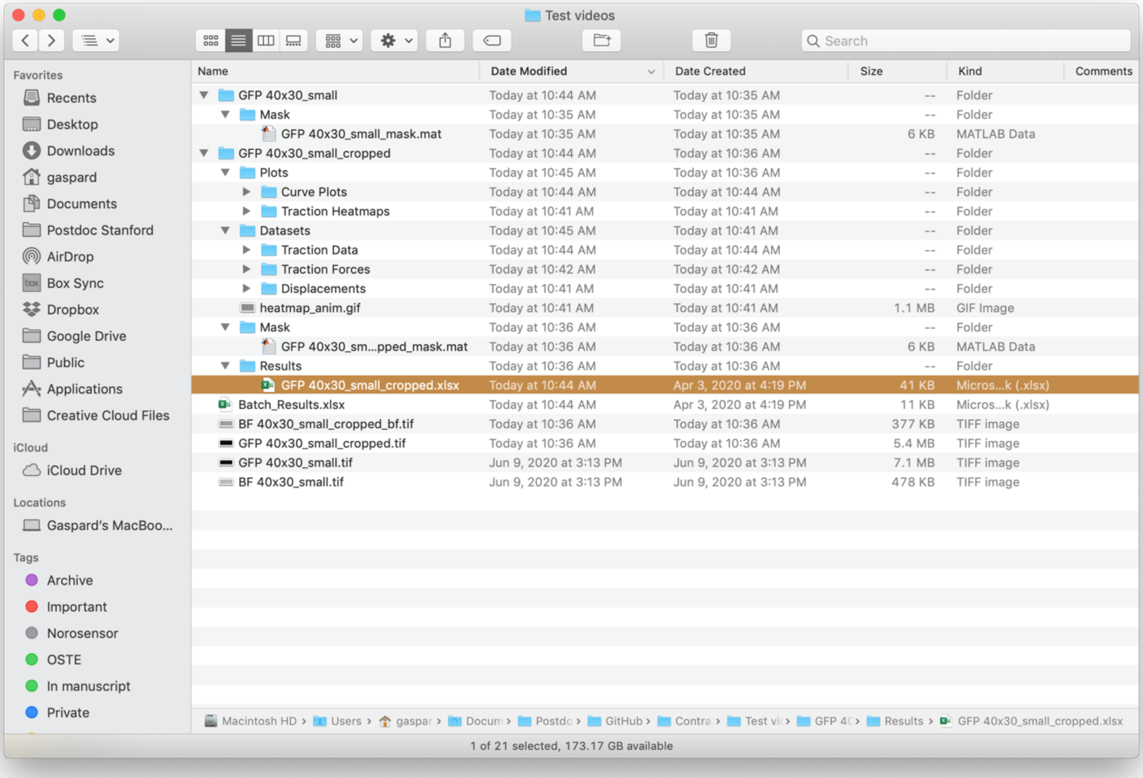

FIGURE 29 FOLDER SUBSTRUCTURE CREATED WITHIN THE FOLDER NAME AFTER THE BEADS VIDEO NAME

REFERENCES

1. Ribeiro, A. J. S. *et al.* Multi-Imaging Method to Assay the Contractile Mechanical Output of Micropatterned Human iPSC-Derived Cardiac Myocytes. *Circulation Research* **120**, 1572–1583 (2017).
2. Pardon, G., Castillo, E. & Pruitt, B. L. A benchmarking model for validation and standardization of traction force microscopy analysis tools. 2020.08.14.250506 Preprint at https://doi.org/10.1101/2020.08.14.250506 (2020).
3. Holenstein, C. N., Lendi, C. R., Wili, N. & Snedeker, J. G. Simulation and evaluation of 3D traction force microscopy. *Comput Methods Biomech Biomed Engin* **22**, 853–860 (2019).
4. Barrasa-Fano, J. *et al.* Advanced in silico validation framework for three-dimensional traction force microscopy and application to an in vitro model of sprouting angiogenesis. *Acta Biomater* **126**, 326–338 (2021).
5. Hansen, P. C. & O'Leary, D. P. The Use of the L-Curve in the Regularization of Discrete Ill-Posed Problems. *SIAM J. Sci. Comput.* **14**, 1487–1503 (1993).

6. Butler JP, Tolić-Nørrelykke IM, Fabry B, Fredberg JJ. Traction fields, moments, and strain energy that cells exert on their surroundings. *American Journal of Physiology-Cell Physiology*. 2002;282(3):C595–C605.
7. Sabass B, Gardel ML, Waterman CM, Schwarz US. High Resolution Traction Force Microscopy Based on Experimental and Computational Advances. *Biophysical Journal*. 2008;94(1):207–220.
8. Jorge-Peñas A, Muñoz-Barrutia A, de-Juan-Pardo EM, Ortiz-de-Solorzano C. Validation tool for traction force microscopy. *Computer methods in biomechanics and biomedical engineering*. 2015;18(13):1377–1385.

9. Sabass B, Gardel ML, Waterman CM, Schwarz US. High resolution traction force microscopy based on experimental and computational advances. *Biophysical Journal*. 2008;94(1):207–220.

10. Pardon G. TFM model. https://zenodo.org/record/3975545 2018:MIT License. DOI: 10.5281/zenodo.3975545.

11. Pardon G., Castillo E., Pruitt B.L., A benchmarking model for validation and standardization of traction force microscopy analysis tools, bioRxiv 2020.08.14.250506; doi:https://doi.org/10.1101/2020.08.14.250506
